# Cerebellar state estimation enables resilient coupling across behavioural domains

**DOI:** 10.1038/s41598-024-56811-x

**Published:** 2024-03-19

**Authors:** Ensor Rafael Palacios, Paul Chadderton, Karl Friston, Conor Houghton

**Affiliations:** 1 University of Bristol, School of Physiology Pharmacology and Neuroscience, Bristol, BS8 1TD UK; 2 UCL, Wellcome Centre for Human Neuroimaging, London, WC1N 3AR UK; 3 University of Bristol, Department of Computer Science, Bristol, BS8 1UB UK

**Keywords:** Cognitive neuroscience, Computational neuroscience, Motor control, Sensorimotor processing

## Abstract

Cerebellar computations are necessary for fine behavioural control and may rely on internal models for estimation of behaviourally relevant states. Here, we propose that the central cerebellar function is to estimate how states interact with each other, and to use these estimates to coordinates extra-cerebellar neuronal dynamics underpinning a range of interconnected behaviours. To support this claim, we describe a cerebellar model for state estimation that includes state interactions, and link this model with the neuronal architecture and dynamics observed empirically. This is formalised using the free energy principle, which provides a dual perspective on a system in terms of both the dynamics of its physical—in this case neuronal—states, and the inferential process they entail. As a demonstration of this proposal, we simulate cerebellar-dependent synchronisation of whisking and respiration, which are known to be tightly coupled in rodents, as well as limb and tail coordination during locomotion. In summary, we propose that the ubiquitous involvement of the cerebellum in behaviour arises from its central role in precisely coupling behavioural domains.

## Introduction

Behaviour, defined as the purposeful engagement of an agent with its environment, is complex, involving interactions and coordination among multiple sensory modalities^1,2^ and motor systems^3,4^. The cerebellum is involved in all aspects of behaviour, ranging from simple stimulus-movement association^5^ to decision making^6,7^, and including coordination of motor systems and effectors^4,8,9^, behaviourally relevant perceptual processing^10^, spatial localisation^11^ and social interactions^12^. Yet, it remains unclear how cerebellar computations support this wide range of functions.

The cerebellum is thought to contribute to extra-cerebellar processing by supplying estimates of behaviourally relevant dynamics, based on internal probabilistic (i.e., generative or forward) models^13–16^. This view is supported by a large amount of empirical evidence^17–19^, showing that neuronal activity in the cerebellum can be read as state estimation^20^. However, the precise nature of the link between cerebellar inference and extra-cerebellar dynamics remains an open question.

Some key cerebellar features are particularly important when looking to understand its function. First, the cerebellum has a relatively simple network architecture, possibly indicating it performs a specific universal or fundamental computation^21^. Next, it receives and reciprocates input from most brain regions^22–25^, making it a locus of convergence for behavioural integration. Lastly, it is primarily involved in learning associations between variables, for example, via cellular plasticity mechanisms^26^. Taken together, these features speak to a high-level, general role of the cerebellum in providing extra-cerebellar regions with precise estimates of how various states interact during behaviour. In other words, because functional behaviour requires dynamic coordination of external and somatic states, we propose that the chief cerebellar function is to efficiently detect, learn and realise context-sensitive interactions among those states, in order to constrain and orchestrate extra-cerebellar dynamics.

This description of cerebellar computations can be formalised within computational neuroscience in many ways. For example, as a Smith predictor, as a forward model, as model predictive control or as an instance of predictive coding^14,27–30^. Here, we will adopt a generic formalism provided by the free energy principle (FEP) that encompasses these accounts. In particular, the FEP furnishes an account of a system’s dynamics, such as neuronal dynamics in the cerebellum, based on minimising prediction errors (a.k.a. surprisal), or, equivalently, maximising model evidence (a.k.a. marginal likelihood)^31,32^.

Crucially, the FEP offers a dual perspective on the dynamics of a system^33^, linking its trajectory in the space of physical—in this case neuronal—states to a trajectory in an implicit encoding space, where each point parameterises a probability distribution over latent states. As a result, an agent interacting with its environment is necessarily a good probabilistic model of this environment, and the action-perception cycle underlying behaviour can be formalised in terms of Bayesian inference; this is known as active inference^32^. This physics of self-organising systems can be applied to neuronal networks^34^, therefore providing a principled way to describe how the cerebellum implements probabilistic models to support behaviour: in essence, under the FEP, the cerebellar architecture defines the form of an implicit generative model, and neuronal dynamics reflect an inference or estimation process under this model. Technically, neuronal dynamics become a gradient flow on variational free energy, where (negative) variational free energy scores the evidence for an agent’s generative model.

The application of the FEP to functional brain architectures generally reduces to identifying the structure and functional form of the generative model that best explains neuroanatomy and neurophysiology. The viewpoint we follow in this paper is that the cerebellum occupies a *deep* or high level in a hierarchical generative model subserving behaviour. This view is supported by the empirical properties of the cerebellum described above, including its widespread connectivity to other brain regions, which speak to a high-level role in contextualising lower, extra-cerebellar levels. This hypothesis is also corroborated by lesion-deficit models, showing that cerebellar lesions lead to deficits (e.g., cerebellar ataxia) that are characterised by a loss of coordination as opposed to behaviour per se. In short, the cerebellum generates the context for, but not the content of, behavioural coordination.

From the perspective of active inference, this means the cerebellum has to recognise the ongoing context—through assimilating ascending prediction errors from extra-cerebellar levels—while, at the same time, supplying empirical priors (c.f., inductive biases) to guide or constrain (i.e., contextualise) dynamics at lower levels via descending predictions to extra-cerebellar levels. Here, the context is encoded by control states, whose inference depends on the co-occurrence or joint evolution of discrete behavioural states—the content. These control states in turn modulate—in a nonlinear fashion—the dynamics of lower (extra-cerebellar) levels. This requires a highly nonlinear extra-cerebellar model, in contrast to a simpler, weakly nonlinear model in the cerebellum, reflected in its simple circuitry. In what follows, we showcase this difference using a linear approximation to cerebellar dynamics and nonlinear (Kuramoto) oscillators at the extra-cerebellar level that are coupled via a cerebellar control state. This kind of architecture undergirds many active inference simulations of coordinated behaviour, and is based on the fundamental separation of context and content in deep generative models of coordination dynamics, see for example^35–37^.

This paper is organised as follows: first, we introduce the FEP, as the theoretical framework for active inference and learning in the brain. Second, we describe how cerebellar models could contribute to active inference by outlining the functional form they could take, and establish a mapping from elements of the model to components of the cerebellar circuitry. We then simulate inference in a cerebellar model both during motor coordination of respiratory and whisker behaviour, and during locomotion-induced coordination of limbs and tail. The results of the simulations reveal that the cerebellum provides a corrective coupling between brain networks to maintain synchronisation in the presence of different types of noise and environmental perturbation. Importantly, these simple behavioural settings evinces the fundamental cerebellar contribution to coordinated behaviour, which can be generalised to all sorts of behavioural paradigms.

## Results

### Neuronal dynamics as inference

Under the FEP, neuronal dynamics are read as an inference process, estimating environmental states $\vartheta$ causing observations $y$. This inference process starts with the specification of a generative model $m$, namely, the joint probability distribution

1 $$\begin{aligned} p(\vartheta , y|m) \end{aligned}$$

between $\vartheta$ and $y$; the form of $m$ is defined by the neuronal architecture and connectivity structure. Then, neuronal inference means that beliefs about environmental states, encoded by neuronal population activity, evolve to match the posterior probability over external states:

2 $$\begin{aligned} \begin{aligned} p(\vartheta |y, m)=\dfrac{p(\vartheta , y|m)}{p(y|m)} \end{aligned} \end{aligned}$$

Hereafter, we omit conditioning on $m$ for simplicity. Since the denominator in this equation, $p(y)$ (a.k.a. model evidence), requires integration over all possible states, it is practically intractable. For this reason, the FEP supposes that the brain performs approximate Bayesian inference in the form of variational Bayes, whereby the negative logarithm of $p(y)$ is approximated by an upper bound called variational free energy ($F$),

3 $$\begin{aligned} -\log p(y)\le F \end{aligned}$$

and neuronal activity encodes approximate beliefs about external states, $q(\vartheta )$ so that

4 $$\begin{aligned} p(\vartheta |y)\approx q(\vartheta ) \end{aligned}$$

where a simple form is assumed for *q*(*y*). In particular, further Gaussian assumptions about random fluctuations imply that neuronal activity encodes the sufficient statistics of $q(\vartheta )$, its mean value or expectation $\mu _{\vartheta }$, which leads to a description of neuronal dynamics in terms of predictive coding or Bayesian filtering^35,38^. In this case, $F$ can be defined as^39^:

5 $$\begin{aligned} F(\mu _{\vartheta }, y)=-\log p(\mu _{\vartheta }, y) \end{aligned}$$

Notably, $F$ can be read as the difference between sensory (or bottom-up) input and (top-down) predictions of that input; namely, the total amount of prediction error incurred during inference. The FEP therefore supposes that the brain solves a difficult inference problem with an easier optimisation procedure, based on minimisation of $F$. Approximate Bayesian inference is plausibly implemented by the brain because $F$ is a function of the model entailed by the network itself, neuronal expectations $\mu _{\vartheta }$, and observations $y$^31,32^. In essence, given a certain model $m$, $F$ is minimised by maximising the probability of co-occurrence of expected states $\mu _{\vartheta }$ and observations $y$. This can be achieved either by changing $\mu _{\vartheta }$, making $q(\vartheta )$ a better approximation of $p(\vartheta |y)$ (see Eq. 21 in methods), or by changing $y$, by acting on external or latent states so that they conform to internal expectations, hence increasing model evidence $p(y)$—a process sometimes known as self evidencing^40^.

### Cerebellar contribution to neuronal inference

The ability of a system to minimise $F$ depends on how well its implicit generative model is able to track environmental dynamics, or alternatively make it match its own dynamics to maximise model evidence. When considering cerebellar computation, one hypothesis is that it improves the accuracy and efficiency of state estimation or inference in other brain regions. In our hypothesis, this is achieved by rapidly contextualising extra-cerebellar inference; that is, the cerebellum may drive or modulate inference in one brain region depending on the specific behavioural context, where the latter corresponds to what is simultaneously being estimated in other brain regions.

Contextualisation is needed because when a system interacts with its environment, behaviour requires coordination; consequently, neuronal dynamics encoding and controlling external and somatic states—within and across brain regions—must be coordinated. The cerebellum appears necessary for fluent coordination, as highlighted by research in the motor and cognitive (e.g., speech) domain^41^. We therefore propose that the cerebellum coordinates information within and across brain regions by means of a general model: simple, thus efficiently estimating states and their associations.

**Figure 1:**
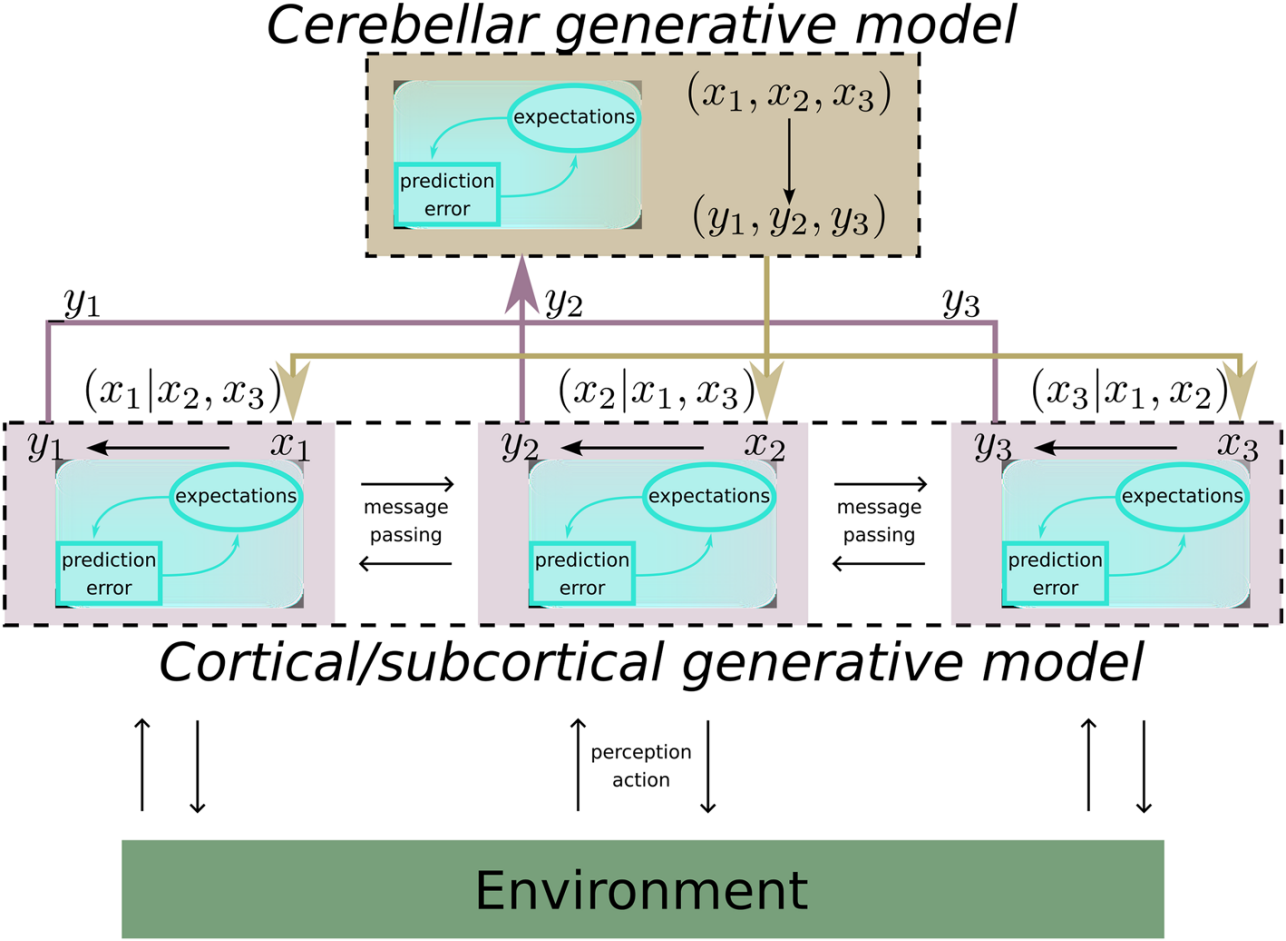
Illustration of cerebellar contribution to active inference in the brain. Extra-cerebellar structures implement a deep generative model of the environment, exemplified by three modules representing different streams of information (violet boxes), all potentially involving hierarchical processing. These modules can underpin discrete but interlinked behaviours, such as whisking and respiration. Within each module, neuronal populations infer or estimate hidden state $x$ from observation $y$ based on forward models (black arrows) of how $x$ generates $y$. The accompanying inference process drives neuronal dynamics to update expectations about $x$ by minimising prediction errors. Furthermore, modules can communicate with each other via within-module message passing, e.g., via predictions and prediction errors. The cerebellum, on the other hand, concomitantly receives and integrates information (observations or prediction errors) from many brain regions, and implements a forward model of how $x$’s interact at lower (extra-cerebellar) levels (black line from all $x$’s to all $y$’s). Then, by providing top-down (empirical prior) constraints on extra-cerebellar inference through its own estimates, the cerebellum can realise expected interactions.

In detail, we entertain the notion that the cerebellum contextualises behaviour by efficiently learning and realising the necessary coordination among states, through predictions that lead to smooth, synchronous and concerted extra-cerebellar inference dynamics. This is illustrated in Fig. 1, where the cerebellar model is placed on top of a modular generative model implemented by extra-cerebellar structures. Crucially, the advantage of this contextual inference relies on how well the cerebellum can bind together brain dynamics, which in turn depends on the underlying generative model.

### The cerebellum: internal model and neuronal dynamics

We suggest that the relatively homogeneous and simple cerebellar architecture implements a linear or weakly nonlinear stochastic state space model. This model defines how the cerebellum extract information from ascending input to estimate behavioural variables and their interactions. This stochastic model includes observations $\varvec{y}$, carrying information from somatic or extra-cerebellar structures to the cerebellum, hidden states $\varvec{x}$, corresponding to behaviourally relevant states that need to be inferred, and hidden causes $\varvec{v}$, which are latent control states mediating interactions among hidden states. (Bold face refers to vectors.) The noise terms $Z$, $W$ and $V$ are normally distributed with covariance $\Sigma _z$ $\Sigma _w$ and $\Sigma _v$. This cerebellar model predicts that $\varvec{y}$ are generated from $\varvec{x}$ through the mapping $g$, and $\varvec{x}$ have dynamics controlled by $\varvec{v}$ via equations of motion $f$; $\varvec{v}$ in turn is unconstrained ($\Sigma _v$ has large variance terms):

6 $$\begin{aligned} \begin{aligned} \varvec{y}&=g(\varvec{x})+Z&\hspace{5ex}Z&\sim \textrm{Normal}(0,\Sigma _z)\\ \dfrac{d\varvec{x}}{dt}=\varvec{x}^\prime&=f(\varvec{x},\varvec{v})+W&\hspace{5ex} W&\sim \textrm{Normal}(0,\Sigma _w)\\ \varvec{v}&=V&\hspace{5ex} V&\sim \textrm{Normal}(0,\Sigma _v) \end{aligned} \end{aligned}$$

A key feature of this model is that it predicts the temporal derivative of hidden states, $\varvec{x}^\prime$, which allows the cerebellum to estimate the trajectory of $\varvec{x}$ from streams of observations $\varvec{y}$. Moreover, the mapping from latent states to observations and equations of motion, defining the predictions of the model, can be approximated with a linear form, conforming with the simple cerebellar architecture:

7 $$\begin{aligned} \begin{aligned} g(\varvec{x})&=\theta _g\varvec{x}\\ f(\varvec{x},\varvec{v})&=-\varvec{x}+\theta _f\varvec{v} \end{aligned} \end{aligned}$$

Equations (6) and  (7) describe an *implicit* cerebellar model of how $\varvec{x}$ and $\varvec{v}$ generate $\varvec{y}$, in the sense that the computations carried out by the underlying neuronal dynamics implement its inversion: the inference process mapping from consequences, $\varvec{y}$, to causes, $\varvec{x}$ and $\varvec{v}$. In more detail, with this particular model in place, state estimation reduces to finding the expected values $\varvec{\mu }_x$, expected motion $\varvec{\mu }_{x^{\prime }}$, and $\varvec{\mu }_v$ that best explain observations $\varvec{y}$. These expectations parameterise cerebellar beliefs about hidden states and are encoded by its population activity. Therefore, given the generative model above, we can specify the requisite estimation equations (i.e., neuronal dynamics) for $\varvec{\mu }_x$, $\varvec{\mu }_{x^{\prime }}$ and $\varvec{\mu }_v$ as follows (please see Eqs. 31–34 for derivations):

8a $$\begin{aligned} \dot{\varvec{\mu }}_x&=\varvec{\mu }_{x}^{\prime }+\kappa _x(\theta _g^T\Pi _z\varvec{\varepsilon }_y-\Pi _w\varvec{\varepsilon }_x) \end{aligned}$$

8b $$\begin{aligned} \dot{\varvec{\mu }}_{x}^{\prime }&=-\kappa _{x^{\prime }}\Pi _w\varvec{\varepsilon }_x \end{aligned}$$

8c $$\begin{aligned} \dot{\varvec{\mu }}_v&=\kappa _{v}(\theta _f^T\Pi _w\varvec{\varepsilon }_x-\Pi _v\varvec{\varepsilon }_v) \end{aligned}$$

where the precision matrices, $\Pi$’s, are the inverse covariance matrices $\Sigma$’s specifying the size of random fluctuations expected by the model in (6) ($\Pi =\Sigma ^{-1}$), $\kappa$’s are learning rates and $\varvec{\varepsilon }$’s are prediction errors:

9 $$\begin{aligned} \begin{aligned} \varvec{\varepsilon }_y&=\varvec{y}-g(\varvec{\mu }_x)\\ \varvec{\varepsilon }_x&=\varvec{\mu }^\prime _x-f(\varvec{\mu }_x,\varvec{\mu }_v)\\ \varvec{\varepsilon }_v&=\varvec{\mu }_v. \end{aligned} \end{aligned}$$

**Figure 2:**
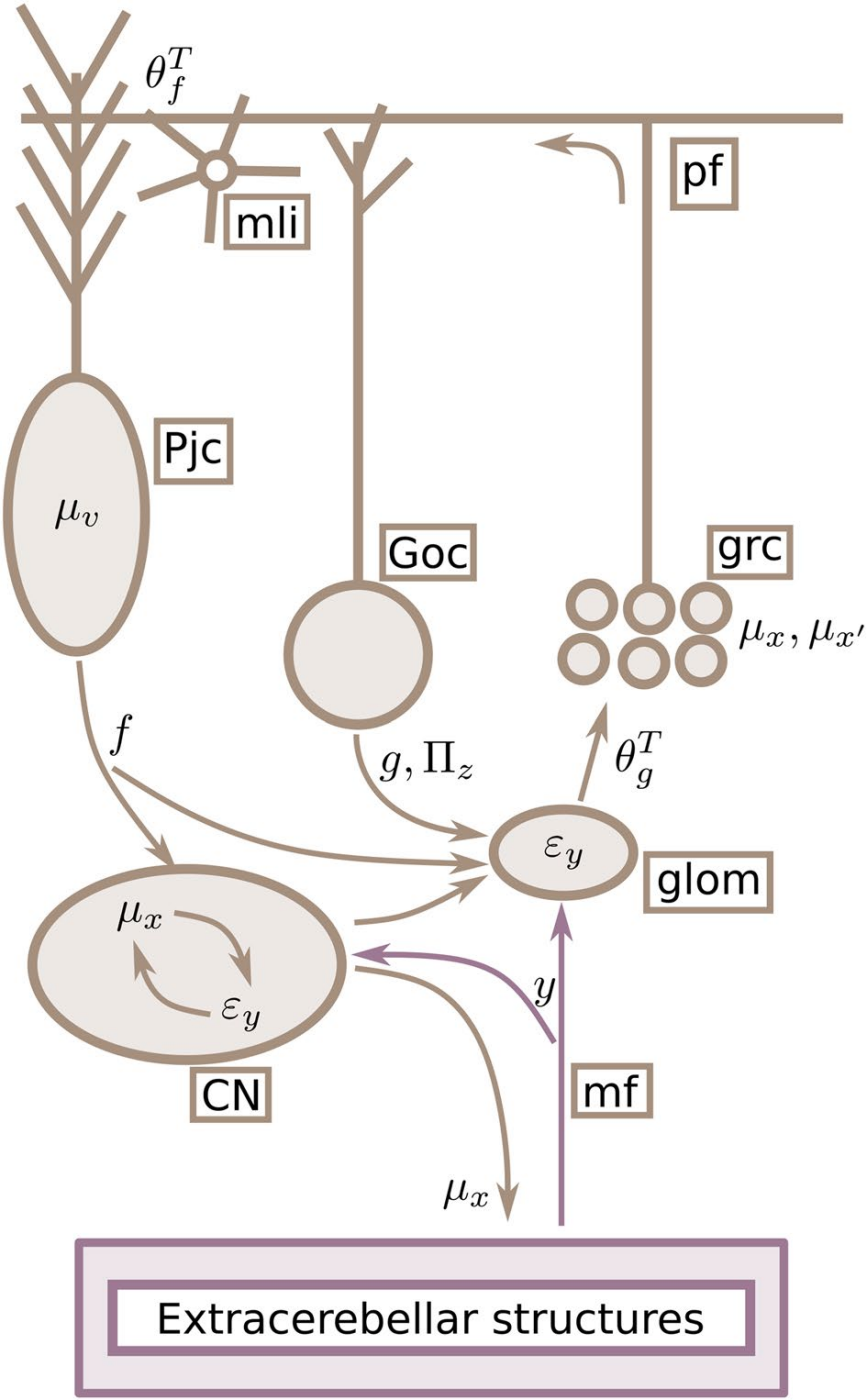
Mapping elements of the model to cerebellar circuitry. Neuronal elements are distinguished from elements of the model by brown boxes. The cerebellum receives observation $y$ from extra-cerebellar structures through **mf**, which drive prediction errors $\varvec{\varepsilon }_y$. This information enters the **CN** as well as the **glom**—specialised structures in the cerebellar cortex where the excitation–inhibition balance (E–I) drives **grc** and **Goc** activity. E–I results from the integration of different inputs, including **Goc**, **CN** and **Pjc** activity. Computationally, this is where descending predictions ($g$, from **Goc**, $f$, from **Pjc** and **CN**) minimise $\varvec{\varepsilon }_y$ and precision ($\Pi _z$, from **Goc**) modulates it. The residual $\varvec{\varepsilon }_y$ then drives **Pjc** and **mli** activity via **pf**, based on the particular connectivity encoded by $\theta _f^T$; this connectivity encodes learned interactions among latent states, which are thus reflected in the dynamics of control states $\mu _{v}$ (**Pjc** activity). These hidden causes are then the source of predictions $f$ modulating **CN** and (directly and indirectly) **grc** activity. This modulation constrains estimates of latent states $\mu _{x}$ according to expectations about their interactions. Finally, these estimates couple back to extra-cerebellar models via **CN** collaterals, updating estimates therein. Mossy fibres **mf**; Purkinje cells **Pjc**; granule cells **grc**; Golgi cells **Goc**; glomeruli **glom**; cerebellar nuclei **CN**; molecular layer interneurons **mli**. .

We can now map the inversion dynamics described in Eqs. (8) and (9) onto specific neuronal components and dynamics of the cerebellum (see Fig. 2). We associate observations $\varvec{y}$ with mossy fibre (**mf**) input, hidden causes $\varvec{\mu }_v$ with Purkinje cell population (**Pjc**) activity, and assume that both hidden states $\varvec{\mu }_x$ and velocities $\varvec{\mu }_{x^\prime }$ are represented by the granule cell population (**grc**). Therefore, **grc** activity reflects not only the instantaneous value of hidden states $\varvec{\mu }_x$, but also their change $\varvec{\mu }_{x^\prime }$, and potentially higher order derivatives, effectively encoding the trajectory of latent states as the coefficients of a Taylor expansion of the path as a function of time.

This temporal property is made explicit in the Eq.  (8a), where changes in expected values of hidden states, $\dot{\varvec{\mu }}_x$, follow $\varvec{\mu }_{x^\prime }$. From a biophysical standpoint, this means that the recent neuronal history impacts current state estimation^42^. Additionally, $\dot{\varvec{\mu }}_x$ also depends on $\theta _g^T\Pi _z\varvec{\varepsilon }_y$ and $\Pi _w\varvec{\varepsilon }_x$: the former equates to new or unexplained information $\varvec{\varepsilon }_y$, based on observations $y$ and predictions $g(\varvec{\mu }_x)$, where $\varvec{\varepsilon }_y$ can be thought of as the net input to **grc** computed within glomeruli (**glom**); a specialised structure containing dendritic and axonal terminals from **grc**, Golgi cells (**goc**) and **mf**.

This net input drives state estimation depending on the connectivity matrix $\theta _g^T$ involving **glom** and **grc**, and is weighted or contextualised by its precision $\Pi _z$ via **goc** inhibition, setting **grc** response gain and threshold^43^; this weighting by $\Pi _z$ controls how much **mf** drives the network, based on the context-dependent precision or relevance of incoming information. On the other hand, the last term, $\Pi _w\varvec{\varepsilon }_x$, incorporates feedback from **Pjc** representing hidden causes $\varvec{\mu }_v$. This contribution may occur either via recurrent inputs from cerebellar nuclei (**CN**)^44,45^—whose dynamics are driven by **mf** and shaped by **Pjc** inhibition—or through direct **Pjc** modulation of cerebellar cortical interneurons^46^ and **grc**^47^.

Next, Eq.  (8b) specifies updates of encoded velocities, $\dot{\varvec{\mu }}_{x}^{\prime }$: these updates depend on predictions $f$ via $\varvec{\varepsilon }_x$, and in absence of (**mf**) input ultimately attract $\varvec{\mu }_{x}$ (**grc** activity) toward zero (enforcing sparsity). Similarly to $\dot{\varvec{\mu }}_x$, updates $\dot{\varvec{\mu }}_{x^\prime }$ are a function of $\varvec{\mu }_v$, and could plausibly rely on the same feedback mechanisms involving **Pjc** and **CN**.

Lastly, the equation for $\dot{\varvec{\mu }}_v$, Eq.  (8c), describes $\varvec{\mu }_v$ (**Pjc** firing) updates that rely on $\varvec{\mu }_x$ and $\varvec{\mu }_{x^\prime }$ (**grc** activity) via $\varvec{\varepsilon }_x$; crucially, how this information is transmitted depends on $\theta _f^T$ , which we associate with the particular connectivity afforded by parallel fibres (**pf**), terminating on molecular layer interneurons (**mli**) and **Pjc**.

Finally, the cerebellar model couples back to extra-cerebellar regions by sending estimates of latent states, $\varvec{\mu }_x$; these are constrained by cerebellar expectations about their interactions or associations, encoded in the **pf** connectivity structure, $\theta _f^T$. This coupling is mediated by **CN**, whose activity also encodes hidden states $\varvec{\mu }_x$, as they are assumed to receive the same information as cerebellar cortex via **mf** collaterals.

### Simulation of motor coordination

We now describe simulations of behavioural coordination, based on the cerebellar-dependent integration of information across multiple brain regions. This integration relies on the fact that the cerebellum learns to infer interactions between sensory modalities, effectors and task contingencies (e.g., preferred outcomes or reward). A key mechanism underlying associative learning is synaptic plasticity at the level of **pf** terminals, as seen in conditioning^48^ and motor adaptation paradigms^49^. Expectations about motor coordination, encoded in the **pf** connectivity matrix ($\theta _g^T$), in turn guide dynamics in extra-cerebellar structures, as the cerebellum provides them with top-down (prior) constraints ensuring optimal behaviour.

**Figure 3:**
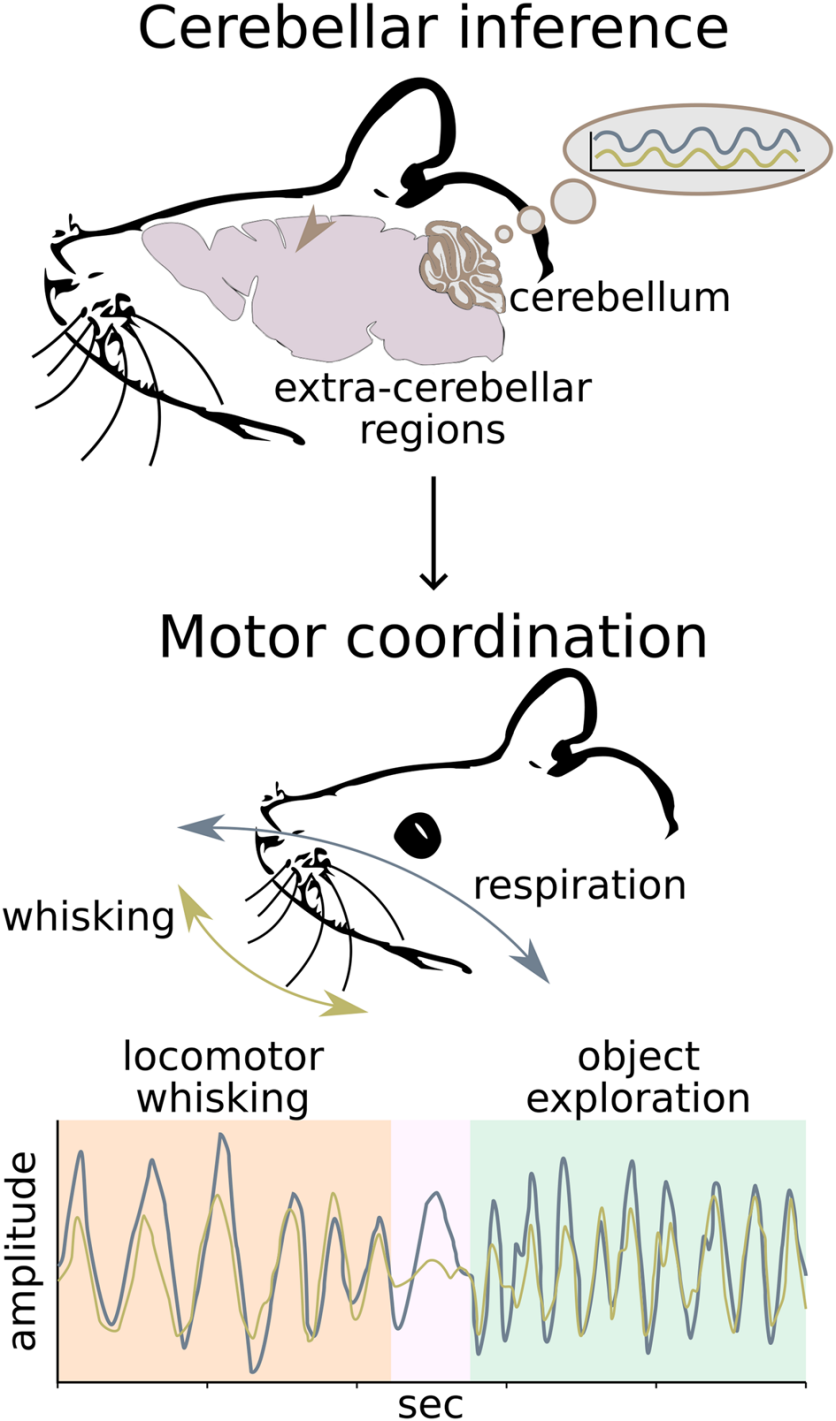
Cerebellar contribution to motor coordination. The cerebellum receives information from extra-cerebellar regions while providing top-down (prior) predictions (violet-brown cycle). Because the cerebellum has learned to expect coordinated (e.g., synchronised) dynamics between behavioural variables, such as respiration (grey line) and whisking (gold line), its predictions can contextualise behavioural domains on each other, by biasing or constraining extra-cerebellar inference. This contextualisation can span different behavioural regimes, here exemplified by a period of locomotor whisking, with low whisking and respiration rates (orange background), and a period of object exploration, with high whisking and respiration rates (green background), separated by a brief period with no whisking (pink background). Ultimately, extra-cerebellar-cerebellar interactions will therefore engender coordination between respiration and whisking.

In particular, we first focus on the coordination of the respiratory and whisker system in mice, a key feature of rodent’s behaviour^3^ (Fig. 3). The production of these behaviours is underpinned by a rich network of interconnected brain structures, including brainstem pattern generators and premotor cortical areas, all of which receive projections from **CN**^4,50,51^. This richness hints at the complexity of extra-cerebellar inference controlling orofacial behaviour, to which the cerebellum contributes by coordinating its various components^4^.

In simulating active inference, we specify both a generative model, implemented by the cerebellum, and a generative process, the extra-cerebellar regions associated with the production of whisking and respiratory patterns. Neuronal dynamics in these regions also reflect an inference process, whereby inference is about latent states related to the whisking and inspiration-expiration cycle (e.g. their amplitude and phase). Extra-cerebellar inference drives motor behaviour, by generating proprioceptive predictions that are realised at the level of motor reflex arcs^52^; however, in this work we do not explicitly simulate extra-cerebellar inference, but approximate it with a stochastic process. Thus, by acting on this process, the cerebellum implicitly tunes the execution of whisking and respiratory behaviour.

In practice, we simulate whisking and respiration cycles with a Kuramoto system, whose output $w$ and $r$ represent somatic states, which could be associated with whisker position and expansion of the rib cage, respectively. In the Kurmoto model the two somatic states are defined by phases

10 $$\begin{aligned} \begin{aligned} r&=\sin {\phi _r}\\ w&=\alpha \sin {\phi _w}. \end{aligned} \end{aligned}$$

The dynamics of these phases is a function of intrinsic angular velocities, $\omega _{w}$ and $\omega _{r}$, and is subject to independent Gaussian noise $Q_w$ and $Q_r$ and offset $\omega _{0}$:

11 $$\begin{aligned} \begin{aligned} \frac{d\phi _{w}}{dt}&=\omega _{w}+k\sin (x_{w}-\phi _{w})+ Q_w&\hspace{3mm}Q_w&\sim \textrm{Normal}(0, \sigma _w) \\ \frac{d\phi _{r}}{dt}&=\omega _{r}+k\sin (x_{r}-\phi _{r})+Q_r&\hspace{3mm}Q_r&\sim \textrm{Normal}(0, \sigma _r) \\ \omega _{r}&=\omega _{w}+\omega _{0}. \end{aligned} \end{aligned}$$

Above we have introduced a term $\alpha$ that, together with $\omega _{w}$ and $\omega _{r}$, is state dependent, that is, it can be changed throughout simulation time, so that the *in silico* mouse can stop or restart whisking, by setting $\alpha$ to 0 or 1, or change the frequency of whisking and respiration, by modifying $\omega _{w}$ and $\omega _{r}$.

The key component of a Kuramoto system is the coupling term; in the standard Kuramoto formulation this is the sine of phase differences, and mediates the synchronisation of the oscillations. Here, in contrast, the coupling terms, $k\sin (x_{w}-\phi _{w})$ and $k\sin (x_{r}-\phi _{r})$ in the whisker and respiration model, relate the phases to $x_{w}$ and $x_{r}$, respectively. These are the corresponding hidden states inferred by the cerebellar generative model, representing extra-cerebellar somatic states $w$ and $r$. The crucial point is that there is no explicit coupling between the two oscillators, instead this coupling emerges from the cerebellar inference that rests on learned expectations about synchrony. In particular, the generative model is the same state space model in (6):

12 $$\begin{aligned} \begin{aligned} \varvec{y}&=g(\varvec{x})+Z&\hspace{3mm} Z&\sim \textrm{Normal}(0,\Sigma _z) \\ \frac{d\varvec{x}}{dt}=\varvec{x}^\prime&=f(\varvec{x},\varvec{v})+W&\hspace{3mm} W&\sim \textrm{Normal}(0,\Sigma _w)\\ \varvec{v}&=V&\hspace{5ex} V&\sim \textrm{Normal}(0,\Sigma _v). \end{aligned} \end{aligned}$$

where $\varvec{y}$, $\varvec{x}$ and $\varvec{v}$ are now associated with whisking and respiration somatic states:

13 $$\begin{aligned} \varvec{y}= \left[ \begin{array}{c} w\\ r \end{array} \right] , \qquad \varvec{x}= \left[ \begin{array}{c} x_{w}\\ x_{r} \end{array} \right] , \qquad \varvec{v}= \left[ \begin{array}{c} v_{w}\\ v_{r} \end{array} \right] . \end{aligned}$$

This formulation assumes that observations $\varvec{y}$—peripheral input originating from the same somatic states $w$ and $r$ returned by the generative process—are mapped linearly from $x_{w}$ and $x_{r}$, which are in turn endowed with dynamics driven by hidden causes $v_{w}$ and $v_{r}$. Notably, cerebellar expectations about $w$-$r$ synchrony can be encoded in the **pf** connectivity $\theta _{f}$:

14 $$\begin{aligned} \begin{aligned} g(\varvec{x})&=\theta _{g}\varvec{x}&\hspace{3mm} \theta _{g}&= \left[ \begin{array}{cc} 1&{}0\\ 0&{}1\\ \end{array} \right] \\ f(\varvec{x},\varvec{v})&=-\varvec{x}+\theta _{f}\varvec{v}&\hspace{3mm} \theta _{f}&= \left[ \begin{array}{cc} 1&{}1\\ 1&{}1 \end{array} \right] . \end{aligned} \end{aligned}$$

These three sets of equations (Eqs. 12–14) define the generative model, whose inversion dynamics (Eqs.  8,  9) we associate with neuronal dynamics in the cerebellum. Cerebellar state estimation, therefore, is about the same whisker and respiratory states that extra-cerebellar models—here approximated by the generative process—are inferring; namely, hidden states $x_{w}$ and $x_{r}$ represent $w$ and $r$. However, the underlying models are different: in the case of the cerebellum, the model is linear and relatively simple, conforming with empirical findings of linear encodings of many task-related parameters^53–55^, whereas in the case of extra-cerebellar models, these can be nonlinear and arbitrarily complex, underlying for example the generation of cyclic patterns and nested sequences^36^.

As such, the equations of motion governing the dynamics of somatic states in extra-cerebellar structures are synthesized in the cerebellum by a linear control of hidden states dynamics $x_{w}^\prime$ and $x_{r}^\prime$ from hidden causes or control states $v_{w}$ and $v_{r}$. Notably, this is where the interaction between whisking and respiration occurs, as estimates of $v_{w}$ and $v_{r}$ (**Pjc** activity) bias through their predictions, $f(\varvec{x},\varvec{v})$, estimates of $x_{w}$ and $x_{r}$—replicated in **CN** activity—which in turn are sent to the generative process where they enter the coupling terms $k\sin (x_{w}-\phi _{w})$ and $k\sin (x_{r}-\phi _{r})$, thus enforcing synchrony between the two oscillators.

**Figure 4:**
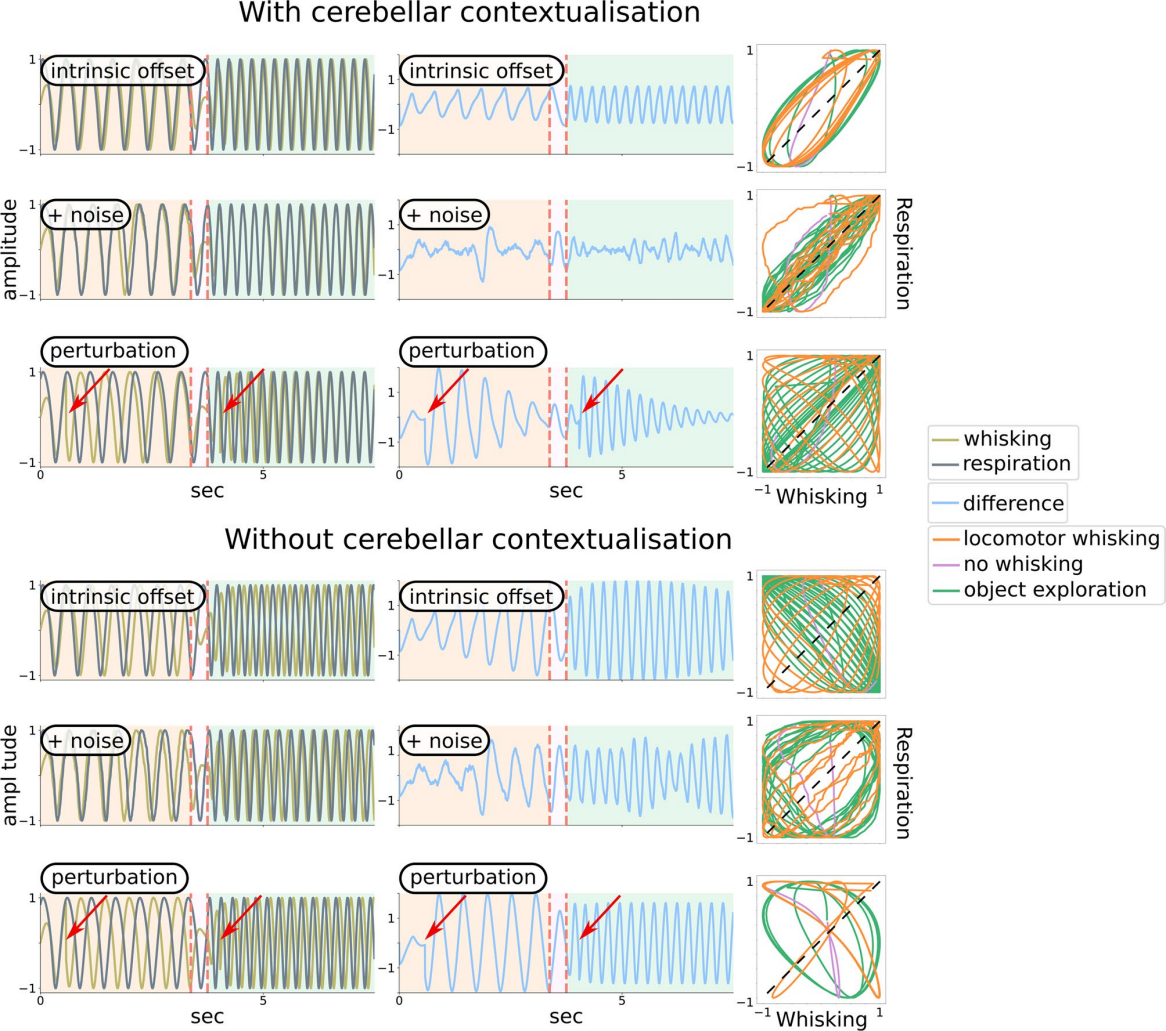
Cerebellar contribution to whisking-respiration coordination (1). Top half (‘With cerebellar contextualisation’): the cerebellar model expects coordination between whisking and respiration; these expectations in turn bias cerebellar estimates of whisking and respiration (please see Fig. 5). The left panels show the evolution of whisking and respiration over time; the middle panels show the evolution of their difference; the right panels show whisking and respiration dynamics in the joint state space together with the synchronisation manifold (straight line). Behavioural state-dependent modes are color-coded, with orange, pink and green background colors (left and middle panel) or lines (right panel) corresponding to low, high whisking/respiration rate and no whisking, respectively; the transition from one mode to another is marked by vertical dotted red lines. There are three simulation conditions: first, whisking and respiration have different intrinsic angular velocities (‘Intrinsic offset’); second, extra-cerebellar dynamics are subject to internal independent noise (‘+ noise’); third, external perturbations are applied to whisking dynamics (the timings of perturbations are indicated by arrows; ‘perturbation’). These conditions apply different types of pressure on behaviour, but cerebellar contextualisation of extra-cerebellar dynamics is able to restore and maintain coordination. Bottom half (‘Without cerebellar contextualisation’): the cerebellum does not expect coordination; therefore it does not contribute to the evolution of whisking and respiration. As a consequence, behaviour quickly becomes chaotic and uncoordinated. Please see video S1 for a simulation combining intrinsic noise with offset in angular velocities.

Having described the setup for modelling motor coordination, we now report the results of the simulations (Fig. 4). In the following, whisking and respiration undergo state-dependent behavioural changes, which exemplify cerebellar-dependent motor coordination in different behavioural regimes. These states include a period of locomotor whisking, characterised by low rates of whisking and respiration; a brief intermediate period during which whisking stops; and a final period of object exploration, characterised by high rates of whisking and respiration (i.e., sniffing).

The results of these numerical studies are read from left to right; first the time evolution of the amplitude or displacement from a reference point of whisker and respiratory somatic states (i.e., $w$ and $r$); second the time evolution of their difference; and third the evolution of whisking and respiration variables in their joint state space, together with the synchronisation manifold (dotted black line).

The top half of Fig. 4 (‘With cerebellar contextualisation’) shows the output of the generative process when the cerebellar model expects whisking-respiration coordination, that is, $\theta _{f}$ is an all-one matrix as in Eq.  (13). Each row is associated with a different simulation condition. On the first row (‘intrinsic offset’), $w$ and $r$ have different angular velocities $\omega _{w}$ and $\omega _{r}$; this offset $\omega _{0}\ne 0$ may be due to central pattern generators having distinct autonomous dynamics. On the second row (‘+ noise’), $w$ and $r$ dynamics are subject to independent Gaussian noise $\textrm{Normal}(0, \sigma _w)$ and $\textrm{Normal}(0, \sigma _r)$. On the third row (‘perturbation’), a perturbation occurs that abruptly changes whisking phase $\phi _{w}$, due to, for example, contact with an external object.

These conditions exemplify different types of (internal and external) sources of behavioural instability, that the cerebellum counteracts efficiently. This is highlighted both in the middle panels, showing that the difference between $w$ and $r$ returns and remains constantly below 1 a.u. (less than half the maximum difference), and in the right panels, where the two variables tend to converge onto the synchronisation manifold (dotted black line) in the joint state space.

**Figure 5:**
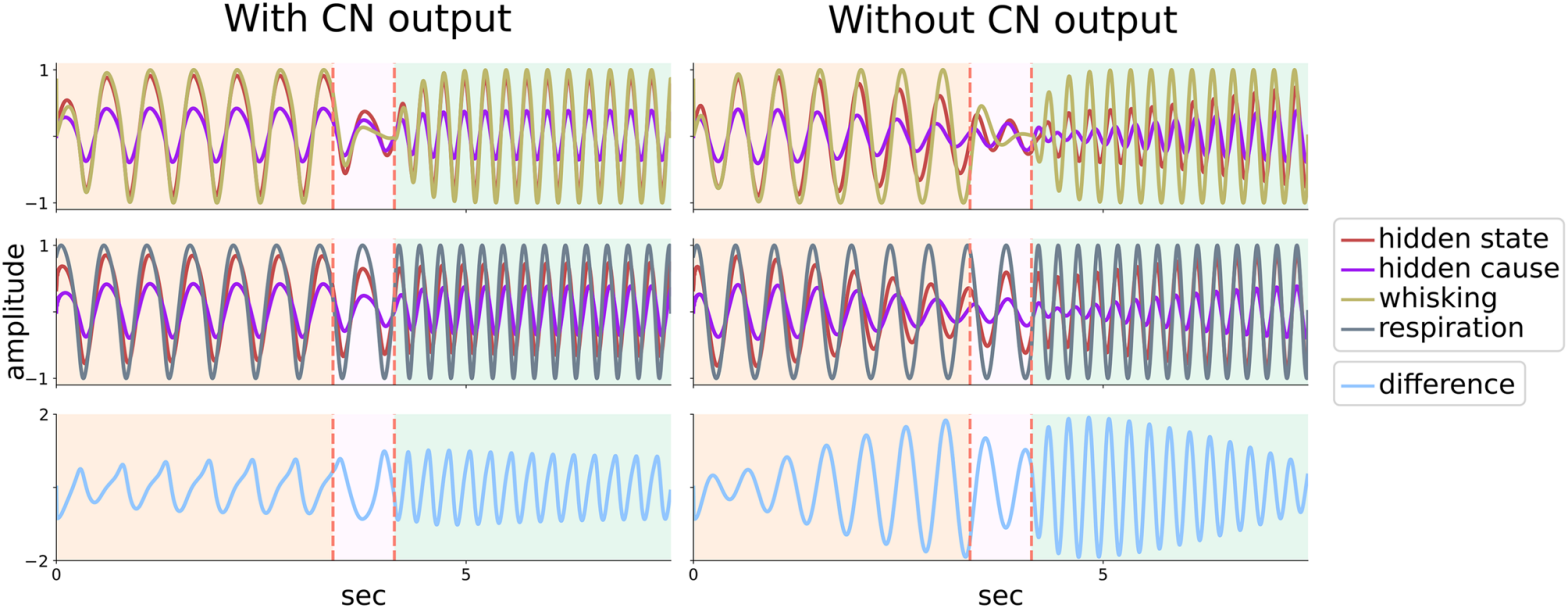
Cerebellar expectations bias state estimation. Simulation results in which the cerebellum expects synchrony between whisking and respiration, whose intrinsic dynamics have different angular velocities. Whisking and respiration variables are split in the top and middle panels, respectively, and are displayed together with cerebellar estimates of the associated hidden states and causes. The bottom panel displays their difference. Left: the cerebellum sends empirical priors via **CN** outputs to extra-cerebellar structures. In this case, estimates about hidden states $x_{w}$ and $x_{r}$ (red lines) closely match real states $w$ and $r$—in virtue of the fact that $w$ and $r$ are constrained towards $x_{w}$ and $x_{r}$—while estimates about hidden causes $v_{w}$ and $v_{w}$ (violet lines) are regular and relatively high in amplitude, which reflects the congruence between expected and observed synchrony between behavioural states. Right: the output of **CN** to extra-cerebellar structures is suspended: now, estimates about hidden states and causes reduce in amplitude the more behaviour desynchronises. This is because there is a friction between predictions and observations, which has an impact on estimates about hidden causes—a function of behavioural synchrony due to expectations in $\theta _{f}$—as well as hidden states—driven by contrasting ascending information and feedback predictions within the cerebellum.

In contrast, the bottom half of Fig. 4 (‘Without cerebellar contextualisation’) shows simulation results in absence of any cerebellar expectations about behavioural coordination; that is, $\theta _{f}$ is an identity matrix. In this case, the two oscillators evolve without any cerebellar-dependent coupling, because cerebellar state estimation is not biased by any expectation about synchrony, and therefore does not realise it. As a consequence, the difference between $w$ and $r$ recursively reaches its peak at 2 a.u., as the two variables are consistently out of phase, circling around the joint state space, far apart from the synchronisation manifold. The (inductive) bias on cerebellar state estimation can be better appreciated by comparing inference in the presence or absence of **cn** outputs, namely, when the cerebellum can or cannot realise expected synchrony (Fig. 5, please see also video S1).

As a second example—dealing with whole-body dynamics—we simulate the coordination of limbs and tail during locomotion. Locomotion relies on coordination between many body parts, which in turn depends on an intact cerebellar cortex: Machado and colleagues showed that in mutant mice with a cerebellar deficit, for instance, pairwise coordination of front and hind limbs on opposite sides is lost, together with symmetric tail dynamics^56^. Here, we model extra-cerebellar neuronal dynamics driving the vertical movement of limbs during the stride cycle:

15 $$\begin{aligned} \begin{aligned} l_{fr}&=\sin {\phi _{fr}}\\ l_{fl}&=\sin {\phi _{fl}}\\ l_{hr}&=\sin {\phi _{hr}}\\ l_{hl}&=\sin {\phi _{hl}}. \end{aligned} \end{aligned}$$

The displacement of the front right ($l_{fr}$), front left ($l_{fl}$), hind right ($l_{hr}$) and hind left ($l_{hl}$) limbs are mapped from their phase $\phi$ in the stride cycle, whose joint dynamics are described by a Kuramoto system, including cerebellar estimates $x$ about limb position:

16 $$\begin{aligned} \begin{aligned} \frac{d\phi _{fr}}{dt}&=\omega +k_1\sin (x_{fr}-\phi _{fr})+ Q_{fr}&\hspace{3mm}Q_{fr}&\sim \textrm{Normal}(0, \sigma _{fr}) \\ \frac{d\phi _{fl}}{dt}&=\omega +k_1\sin (\phi _{fr}-\phi _{fl})+k_2\sin (x_{fl} -\phi _{fl})+Q_{fl}&\hspace{3mm}Q_{fl}&\sim \textrm{Normal}(0, \sigma _{fl}) \\ \frac{d\phi _{hr}}{dt}&=\omega +k_1\sin (\phi _{fr}-\phi _{hr})+k_2\sin (x_{hr} -\phi _{hr})+Q_{hr}&\hspace{3mm}Q_{hr}&\sim \textrm{Normal}(0, \sigma _{hr}) \\ \frac{d\phi _{hl}}{dt}&=\omega +k_1\sin (\phi _{fr}-\phi _{hl})+k_2\sin (x_{hl} -\phi _{hl})+Q_{hl}&\hspace{3mm}Q_{hl}&\sim \textrm{Normal}(0, \sigma _{hl}) \end{aligned} \end{aligned}$$

**Figure 6:**
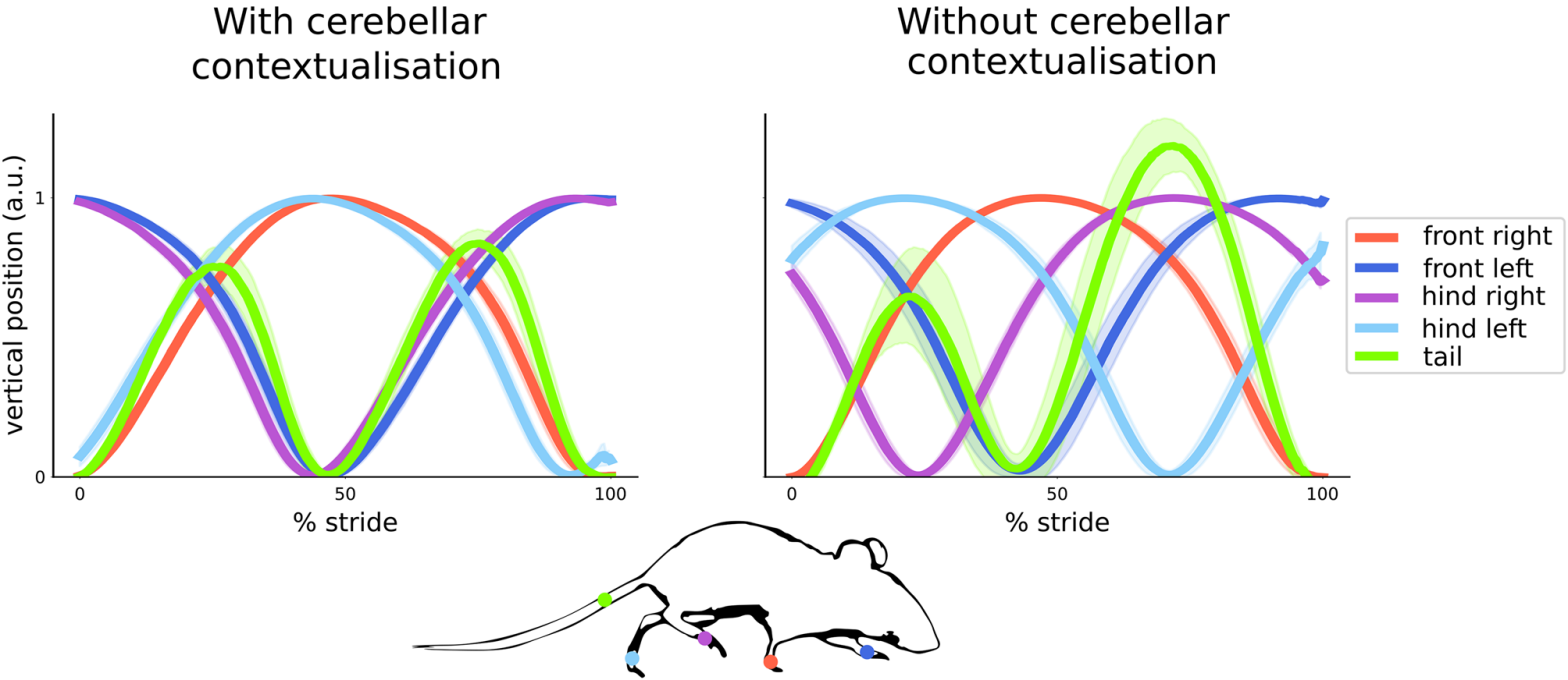
Cerebellar-dependent coordination of limb and tail movements during locomotion. Left (‘With cerebellar contextualisation’): average vertical displacement of the front right, front left, hind right and hind left limbs as well as of the tail during locomotion across 14 strides. Shaded area denotes the standard deviation. In the presence of cerebellar expectations of inter-limb coordination, the front right and front left limbs are phase locked with the hind left and hind right limbs, respectively, and the two pairs of limbs have opposite phase. This results in the pairwise coordination of limb vertical displacement. At the same time, tail movements, which are modelled as a passive consequence of limb dynamics, symmetrically follows the dynamics of the two pairs of limbs. Right (‘Without cerebellar contextualisation’): in the absence of cerebellar control of limb dynamics there is no front-hind limb coordination, as each limb moves out of phase compared to the others, and tail movements become asymmetric.

The generative process imposes a phase shift of $\pi , \frac{3}{2}\pi$ and $\frac{1}{2}\pi$ to $l_{fl}$, $l_{hr}$ and $l_{hl}$ compared to the $l_{fr}$, through the coupling terms $k_2\sin (\phi _{hr}-\phi )$. This bias maximises the time during which at least one paw is touching the ground, and characterises locomotion in absence of cerebellar contributions to whole-body coordination in mice; individual limb dynamics, in contrast, are indistinguishable from those described in healthy animals^56^ (Fig. 6, right).

On the other hand, when cerebellar compensatory input, $k_1\sin (x-\phi )$, are present, $l_{fr}$ and $l_{hl}$ are phase-locked, as do $l_{fl}$ and $l_{hr}$, and the two pairs are anti-correlated (Fig. 6, left). Compensatory input from the cerebellum rest on a generative model (a state space model as in Eq.  12) that holds expectations about inter-limb coordination in the **pp** connectivity matrix $\theta _{f}$:

17 $$\begin{aligned} \begin{aligned} g(\varvec{x})&=\theta _{g}\varvec{x}&\hspace{3mm} \theta _{g}&= \left[ \begin{array}{cccc} 1&{}0&{}0&{}0\\ 0&{}1&{}0&{}0\\ 0&{}0&{}1&{}0\\ 0&{}0&{}0&{}1 \end{array} \right] \\ f(\varvec{x},\varvec{v})&=-\varvec{x}+\theta _{f}\varvec{v}&\hspace{3mm} \theta _{f}&= \left[ \begin{array}{cccc} 1&{}-1&{}-1&{}1\\ -1&{}1&{}1&{}-1\\ -1&{}1&{}1&{}-1\\ 1&{}-1&{}-1&{}1 \end{array} \right] . \end{aligned} \end{aligned}$$

where

18 $$\begin{aligned} \varvec{x}= \left[ \begin{array}{c} x_{fr}\\ x_{fl}\\ x_{hr}\\ x_{hl} \end{array} \right] , \qquad \varvec{v}= \left[ \begin{array}{c} v_{fr}\\ v_{fl}\\ v_{hr}\\ v_{hl} \end{array} \right] . \end{aligned}$$

Finally, tail movements can be modelled as a passive consequence of limb dynamics^56^:

19 $$\begin{aligned} \begin{aligned} t&=\sin {\phi _{fr}} + \sin {\phi _{fl}}\\ \end{aligned} \end{aligned}$$

Notably, in the presence of cerebellar coordination, tail excursions on the vertical axis ($t$) are symmetric with respect to the dynamics of the two pairs of limbs (Fig. 6), which may improve balance and movement efficiency. Conversely, this symmetry is disrupted in the absence of cerebellar input. In other words, in our model, optimal tail movement during locomotion emerges from the cerebellar control and correction of limb dynamics.

In summary, these simulations illustrate how synchronisation of whisking and respiration is dependent on the cerebellar generative model: when this model holds expectations about motor coordination, it is able to orchestrate extra-cerebellar dynamics during various behavioural regimes (e.g., locomotor whisking and object exploration), despite the presence of different intrinsic rhythms and sources of noise. On the other hand, this cerebellar contribution is absent in the case of a naive model, namely, before learning or after lesion of **pf**.

## Discussion

The cerebellum is a brain structure that plays a key role in the execution of behaviour. Notably, while it is not necessary for the production of behaviour, it is crucial for its coordination, fluency and resilience^56^. At any given time, the cerebellum is thought to generate estimates about current and future states, such as upcoming limb positions, environmental dynamics, and mental states^16^. However, the general principle guiding the generation of these neuronal representations, and their use in fine tuning behaviour, is still unclear. Here, we formalised the idea that the key cerebellar function is to estimate how different variables interact, and to bind them together to produce coordinate behaviour. In particular, we have described a general cerebellar model underlying behavioural coordination, and outlined how it could be implemented biologically. This model is simple, in accordance with the crystal-like cerebellar architecture, while general, compatible with the wide range of behavioural functions the cerebellum is involved in. As paradigmatic examples, we have simulated whisking-respiration and limbs-tail coordination, two processes that showcase the general but crucial contribution of cerebellar associative learning to behaviour.

One key challenge, faced by biological systems, is to constantly adapt behaviour to changing conditions and perturbations. This adaptation requires biological systems to learn new behavioural contexts, namely, new interactions between somatic and external states. For example, in the case of the vestibulo-ocular reflex, adjusting its gain in response to experimental manipulation would require tuning expectations about the interaction strength between visual and vestibular variables. In the present model, this would entail changing the connectivity matrix involving the **pf**, $\theta _{f}^T$.

**Figure 7:**
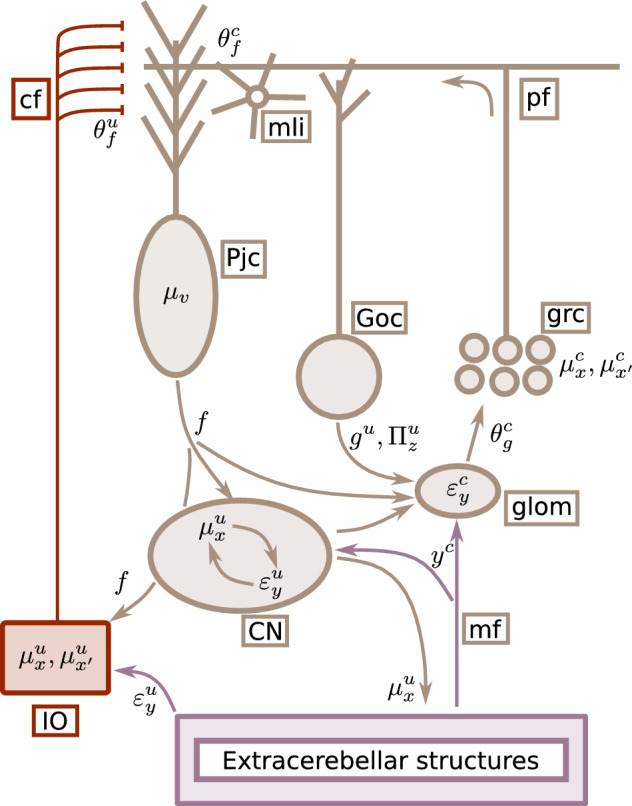
Olivo-cerebellar circuitry. Mapping of elements of the model for a conditioning paradigm to elements of the olivo-cerebellar circuitry. The mossy fibre pathway is the same as in Fig. 2, but here it is exclusively concerned with the estimation of the conditioned stimulus (c superscript). The inferior olive-climbing fiber pathway, instead, is concerned with the estimation of the unconditioned stimulus (u subscript), also based on a state space model (Eq. 23). In practice, the inferior olive (**IO**) could receive prediction errors $\varepsilon _{y}^{u}$ about the unconditioned stimulus from extra-cerebellar structures, as shown in Fig. 1—illustrating message passing between neuronal structures. Meanwhile, the **IO** would also receive predictions $f$ about the motion of unconditioned states, $\mu _{x^{\prime }}^{u}$, from **CN**-mediated **Pjc** input. Together, error and prediction input would drive expectations encoded by **IO** neurons about the unconditioned stimulus, $\mu _{x}^{u}, \mu _{x^{\prime }}^{u}$. Notably, the comparison between predicted, $f$, and expected motion of the unconditioned state, $\mu _{x^{\prime }}^{u}$, encoded by error units $\varepsilon _{x}$ in the **IO**, would drive **cf** activity and subsequent updates of expectations about the control state, $\mu _{v}$, encoded in the **Pjc** population. In a conditioned paradigm, the climbing fibre pathway described above is responsible for the estimation of the unconditioned stimulus that drives baseline behavioural response pattern in extra-cerebellar structures. Following learning, the presentation of the conditioned stimulus becomes associated with the unconditioned stimulus, and can elicit a response on its own, called conditioned response. Associative learning in the cerebellum involves changes in the **pf** connectivity matrix, $\theta _{f}^{c}$, and, similarly to inference, it complies with the free energy minimisation imperative, but on a slower time scale: thus, learning can be specified with a gradient descent on free energy with respect to $\theta _{f}$. In this specific case, for example, $\theta _{f}^{c}$ would increase in value because the cerebellar model that best explains **IO** and **mf** input is the one which expects the control state $\mu _{v}$, indicating the behavioural context, to generate both unconditioned, $\mu _{x}^{u}$, and conditioned states, $\mu _{x}^{c}$.

Our model focuses on the **mf** pathway, but climbing fibre (**cf**) inputs are also crucial for estimating and learning interactions among behavioural variables. Similarly to **mf**, **cf** carry a wide range of sensory, motor and cognitive afferents^7,57–60^, which can also be regarded as prediction errors, updating **Pjc** estimates of the ongoing behavioural context. Importantly, **cf** input are particularly informative about changes in behavioural contingencies (e.g. presence of an unconditioned stimulus in conditioning paradigms^28^), in the sense that they drive learning of new interactions or associations between states (e.g. conditioned stimulus and unconditioned responses), by triggering changes in the **pf** connectivity (please see Fig. 7). Thus, behavioural adaptation rests on fine tuning of cerebellar expectations by **cf** input.

Practically, cerebellar state estimation and the underlying neuronal dynamics comply with the FEP, which explains the dynamics of biological systems from first physics principles (e.g., a principle of stationary action)^32,33^. Under the FEP, neuronal networks implicitly invert probabilistic models of their environment, such that physical dynamics of neuronal (internal) states are equipped with belief dynamics in the space of probability distributions over (environmental) states. This licenses a formal link between cerebellar networks^21^ and their internal models^15,16^. In this formulation, the cerebellum performs Bayesian filtering based on a state space model, which generalises prominent theories of the cerebellum as a state estimator or Kalman filter^13,14,20^.

In active inference, the generative model implemented by the brain is dynamic, meaning that forward models are an integral part of sensorimotor control. This effectively allows state estimation throughout the brain to compensate for delays incurred by in neuronal message passing between peripheral and central nervous system, or between brain areas. In other words, because both action and perception are the product of inference, the delay affecting sensory feedback or prediction errors are accounted for by predictions about the dynamics of proprioceptive and external states. Please see^61^ for a worked example in the oculomotor system.

The cerebellar model is an integral part of the overarching model that is the nervous system. The key question is therefore: what is the specific cerebellar contribution to inferential processes in the brain? We proposed that its key function is to learn and infer how dynamics across the brain are associated, and to enact these associations by constraining (i.e., contextualising) them via empirical priors. This has several implications that need to match empirical evidence. First, it requires precise representations of behavioural dynamics. Indeed, the cerebellum matches dynamics in extra-cerebellar structures, and accurately represents all kinds of variables, including motor, sensory, somatic, autonomic and cognitive states, task-contingencies (e.g., reward signals) and general motor activation^4,62–65^.

Second, cerebellar state estimation relies on (approximately) linear computations, which conform with empirical findings of linear encoding of many task-related variables^53–55^. Nevertheless, this model also accounts for the often neglected recurrent connectivity within the cerebellum, involving feedback loops from **Pjc** and **CN** back to **grc**, **Goc** and other interneurons^44–47^. Moreover, various network and cellular mechanisms, including resonance in the **grc** layer^26^, intrinsic excitability of **Pjc**^66^ and rebound bursting of **CN** neurons^67^ may also allow the cerebellar model to account for some forms of nonlinear interactions between variables, which could be introduced by using nonlinear functions $g$ and $f$^68^. In this case, the cerebellum could model hidden states as the product of the nonlinear convolution of hidden causes, and ensuing state estimation would result in the nonlinear deconvolution of extra-cerebellar input^69^; such a cerebellar model, in turn, could be particularly appropriate when dealing with behavioural contexts where more complex interactions between behaviourally-relevant states are in play.

Finally, cerebellar outputs enter extra-cerebellar inference as empirical priors, constraining or modulating neuronal dynamics to conform with its own predictions. This places the cerebellum at a high level in the brain hierarchy, in reciprocal communication with virtually all brain structures, including peripheral networks^70^, subcortical structures^71,72^, and most cortical areas^29,73^. Importantly, cerebellar outputs do not substitute the content of inference in extra-cerebellar structures, but rather refines it based on information about conditional dependencies among behaviourally relevant variables. This can account for the cerebellar widespread contributions to behaviour, involving preparatory activity underlying decision making^7^ and motor initiation^6^, calibration of ongoing movement trajectories^70,74^, precise refinement of timing, amplitude and sequencing of conditioned responses^5^, reflexes^75^, as well as mental (e.g., speech) processing^41^, and more in general coordination of behaviour in time and space^11,76^. Crucially, all these contributions rely on learned associations between task-relevant variables.

## Methods

### Neuronal dynamics as inference

In Bayesian inference, we seek the posterior distribution over unknown states $\vartheta$, given some observations $y$ and a model $m$ of how these observations have been generated:

20 $$\begin{aligned} p(\vartheta |y, m)=\dfrac{p(y|\vartheta , m)p(\vartheta |m)}{p(y|m)}. \end{aligned}$$

Finding the posterior is generally intractable due to the calculation of the marginal likelihood or normalisation constant (a.k.a. model evidence) $p(y|m)$. Therefore, it has been suggested that the brain approximates Bayesian inference using variational Bayes, an optimisation procedure that allows us to find a variational or recognition density over environmental states, $q(\vartheta )$, which approximates the true posterior $p(\vartheta |y)$: this enables to solves a difficult inference problem with an easier optimisation procedure. This optimisation relies on the the minimisation of the Kullback-Liebler (KL) divergence between the proposed and true posterior density. Importantly, the KL divergence can be expressed as a variational free energy (F)^39^. Leaving out the $m$ for clarity, we substitute from the Bayes law, Eq. (20)

21 $$\begin{aligned} \begin{aligned} D_{kl}(q(\vartheta )|p(\vartheta |y))&=\int q(\vartheta ) \log {\dfrac{q(\vartheta )}{p(\vartheta |y)}}d\vartheta \\&=F+\log p(y) \end{aligned} \end{aligned}$$

where

22 $$\begin{aligned} F(q(\vartheta ), y)=\int q(\vartheta )\log \dfrac{q(\vartheta )}{p(\vartheta , y)}d\vartheta . \end{aligned}$$

$F$ depends on the generative model $m$, since $p(\vartheta ,y)$ does, as does the recognition density $q(\vartheta )$. The sufficient statistics (e.g., mean or expectation) of $q(\vartheta )$ are assumed to be encoded by the brain. The equation for the KL divergence, Eq. (21), shows that minimising $F$ by changing $q(\vartheta )$ automatically reduces $D_{kl}$, because $\log p(y)$ does not depend on $q(\vartheta )$. Thus, minimising the free energy $F$ makes the recognition density, $q(\vartheta )$ a good proxy for $p(\vartheta |y)$.

This leaves open the question of what form $q(\vartheta )$ takes; under the Laplace approximation it is restricted to a Gaussian distribution. Thus, in this picture, the brain encodes $q(\vartheta )$ through the sufficient statistics $\mu$ and $\xi$, the mean and variance respectively, of a Gaussian distribution $q(\vartheta ;\mu ,\xi )$^38^, effectively constraining beliefs about hidden or latent states to be normally distributed. Under the Laplace assumption, the variance of the recognition density that minimises free energy is an analytic function of the expectation; this means that the brain only needs to encode the expected value $\mu$ of these beliefs in order to minimise $F$.

### State space models

The functional form of $F$ is specified by $m$, which is, in this work, a stochastic state space model with first-order generalised motion (bold face refers to vectors)^38^:

23 $$\begin{aligned} \begin{aligned} \varvec{y}&=g(\varvec{x})+Z&\hspace{5ex}Z&\sim \textrm{Normal}(0,\Sigma _z)\\ \dfrac{d\varvec{x}}{dt}=\varvec{x}^\prime&=f(\varvec{x},\varvec{v})+W&\hspace{5ex} W&\sim \textrm{Normal}(0,\Sigma _w)\\ \varvec{v}&=V&\hspace{5ex} V&\sim \textrm{Normal}(0,\Sigma _v). \end{aligned} \end{aligned}$$

This model assumes the existence of two hidden or latent states, $\varvec{v}$ and $\varvec{x}$, which bring about observations $\varvec{y}$; $\varvec{v}$ can be thought of as the control or exogenous input to a system and $\varvec{x}$ as the state of a system whose temporal dynamics $\varvec{x}^{\prime }$ are a function of itself and the control states $\varvec{v}$. On the other hand, $Z$ and $W$ are noise terms affecting the mapping $g$ from $\varvec{x}$ to $\varvec{y}$ and equations of motion for $\varvec{x}$; $V$ is a noise term with high variance, indicating a noninformative prior over $\varvec{v}$. With this model, the free energy can then be decomposed as (omitting some terms for clarity):

24 $$\begin{aligned} \begin{aligned} F(\varvec{\mu }, \varvec{y})&=-\log p(\varvec{\mu }, \varvec{y}) =-\log p(\varvec{\mu }_x, \varvec{\mu }_v, \varvec{y})\\&=-\log p(\varvec{y}|\varvec{\mu }_x)-\log p(\varvec{\mu }_x|\varvec{\mu }_v)-\log p(\varvec{\mu }_v) \end{aligned} \end{aligned}$$

and, using the model, as described in Eq. (23), these log probabilities can be written as:

25 $$\begin{aligned} \begin{aligned} \log p(\varvec{y}|\varvec{\mu }_x)&=-\dfrac{1}{2}(\varvec{y}-g(\varvec{\mu }_x))^T\Sigma _z^{-1}(\varvec{y} -g(\varvec{\mu }_x))-\dfrac{1}{2}\log |\Sigma _z|\\ \log p(\varvec{x}|\varvec{v})&=-\dfrac{1}{2}(\varvec{\mu }^\prime _x-f(\varvec{\mu }_x,\varvec{\mu }_v))^T \Sigma _w^{-1}(\varvec{\mu }^\prime _x-f(\varvec{\mu }_x,\varvec{\mu }_v))-\dfrac{1}{2}\log |\Sigma _w|\\ \log p(\varvec{v})&=-\dfrac{1}{2}\varvec{\mu }_v^T\Sigma _v^{-1}\varvec{\mu }_x-\dfrac{1}{2}\log |\Sigma _v|\end{aligned} \end{aligned}$$

which in turn gives the following functional form:

26 $$\begin{aligned} F(\varvec{\mu _\vartheta }, \varvec{y})=\dfrac{1}{2}\varvec{\varepsilon }_y^T\Pi _z\varvec{\varepsilon }_y-\dfrac{1}{2}\log |\Pi _z|+\dfrac{1}{2}\varvec{\varepsilon }_x^T\Pi _w\varvec{\varepsilon }_x-\dfrac{1}{2}\log |\Pi _w|+\dfrac{1}{2}\varvec{\varepsilon }_v^T\Pi _v\varvec{\varepsilon }_v-\dfrac{1}{2}\log |\Pi _v|\end{aligned}$$

where we have used the precision matrix in place the covariance matrix ($\Pi =\Sigma ^{-1}$) and the auxiliary variables $\varvec{\varepsilon }$ to indicate the prediction error terms:

27 $$\begin{aligned} \begin{aligned} \varvec{\varepsilon }_y&=\varvec{y}-g(\varvec{\mu }_x)\\ \varvec{\varepsilon }_x&=\varvec{\mu }^\prime _x-f(\varvec{\mu }_x,\varvec{\mu }_v)\\ \varvec{\varepsilon }_v&=\varvec{\mu }_v\\ \end{aligned} \end{aligned}$$

where

28 $$\begin{aligned} \begin{aligned} g(\varvec{\mu }_x)&=\theta _g\varvec{\mu }_x\\ f(\varvec{\mu }_x,\varvec{\mu }_v)&=-\varvec{\mu }_x+\theta _f\varvec{\mu }_v. \end{aligned} \end{aligned}$$

It is proposed that neuronal dynamics can be understood as minimising $F$. This can be achieved by specifying their temporal evolution as a gradient descent on $F$:

29 $$\begin{aligned} \begin{aligned} \dot{\varvec{\mu }}=\varvec{\mu }^\prime -\varvec{\kappa }_{\mu }\dfrac{dF(\varvec{\mu },\varvec{y})}{d\mu } \end{aligned} \end{aligned}$$

where $\varvec{\kappa }$ are rate constants. It is important to note that in this gradient descent scheme, the term updating expectations about beliefs encoded by neuronal dynamics, $\dot{\varvec{\mu }}$, is distinct from the motion of those expectations, $\varvec{\mu }^\prime$; that is, the motion of the expectations is distinct from expected motion. The two equate only when $F$ is minimised^38^. The equation of motion, Eq. (29), describes the general form of *recognition dynamics* underlying perception, that is, the dynamics of neuronally encoded states which minimise $F$. Alternatively, minimisation of $F$ can be achieved by changing the state of the environment through action $a$, so that observations conform to beliefs^52^. Action dynamics can be specified with a gradient descent on $F$:

30 $$\begin{aligned} \begin{aligned} \dot{a}&=-\kappa _a\dfrac{dF(\varvec{\mu },\varvec{y})}{da}\\ \dfrac{F(\varvec{\mu },\varvec{y})}{da}&=\dfrac{d\varvec{y}}{da}\dfrac{dF(\varvec{\mu },\varvec{y})}{d\varvec{y}}. \end{aligned} \end{aligned}$$

In this formulation, action $a$ does not enter in the definition of $F(\varvec{\mu },\varvec{y})$, because the brain does not entertain any explicit belief about $a$ in its model $m$ about the environment. However, the brain possesses an *inverse model* of how $a$ acts on the environment and changes $\varvec{y}$: this could be implemented for example by a cascade of belief propagation down to the level of arc reflexes in the spinal cord, hardwired to satisfy expectations about limb position by producing the appropriate pattern of muscle contractions^52^. In the present work, because of the way we couple the cerebellum to extra-cerebellar structures through estimates of $x$, we do not deal with $a$ explicitly. As such, we only focus on the recognition dynamics for $\varvec{x}$, $\varvec{x}^{\prime }$, $\varvec{v}$ based on the internal energy, Eq. (26). This gives dynamical equations for the means:

31 $$\begin{aligned} \begin{aligned} \dot{\varvec{\mu }}_x&=\varvec{\mu }_{x}^{\prime }-\kappa _x\dfrac{F(\varvec{\mu },\varvec{y})}{d\varvec{\mu }_x}\\ \dot{\varvec{\mu }}_{x}^{\prime }&=-\kappa _{x^{\prime }}\dfrac{F(\varvec{\mu },\varvec{y})}{d\varvec{\mu }_{x}^{\prime }}\\ \dot{\varvec{\mu }}_v&=-\kappa _{v}\dfrac{F(\varvec{\mu },\varvec{y})}{d\varvec{\mu }_v} \end{aligned} \end{aligned}$$

Here the expected motion $\varvec{\mu }_x^{\prime }$ only enters the recognition dynamics of $\varvec{\mu }_x$, because for $\varvec{\mu }_{x}^{\prime }$ there is no expected motion, that is, there is no $\varvec{\mu }_x^{\prime \prime }=d\varvec{\mu }_x^{\prime }/dt$, and $v$ does not have any dynamics. Finally, the partial derivatives in equations of motions for the means, Eq. (31), can be expanded into equations for the rate of change of $F(\varvec{\mu },\varvec{y})$ with respect to $\varvec{\mu }_x$:

32 $$\begin{aligned} \begin{aligned} \dfrac{F(\varvec{\mu },\varvec{y})}{d\varvec{\mu }_x}&=\dfrac{1}{2}\left( \dfrac{d[\varvec{\mu }_y-\theta _g\varvec{\mu }_x]^T}{d\varvec{\mu }_x}2\Pi _z\varvec{\varepsilon }_y\right) +\dfrac{1}{2}\left( \dfrac{d[\varvec{\mu }_{x^{\prime }} -(-\varvec{\mu }_x+\theta _f\varvec{\mu }_v)]^T}{d\varvec{\mu }_x}2\Pi _w\varvec{\varepsilon }_x\right) \\&=-\theta _g^T\Pi _z\varvec{\varepsilon }_y+\Pi _w\varvec{\varepsilon }_x \end{aligned} \end{aligned}$$

with respect to $\varvec{\mu }_{x^{\prime }}$:

33 $$\begin{aligned} \dfrac{F(\varvec{\mu },\varvec{y})}{d\varvec{\mu }_{x^{\prime }}}=\dfrac{1}{2}\left( \dfrac{d[\varvec{\mu }_{x^{\prime }} -(-\varvec{\mu }_x+\theta _f\varvec{\mu }_v)]^T}{d\varvec{\mu }_{x^{\prime }}}2\Pi _w\varvec{\varepsilon }_x\right) =\Pi _w\varvec{\varepsilon }_x \end{aligned}$$

and with respect to $\varvec{\mu }_v$:

34 $$\begin{aligned} \dfrac{F(\varvec{\mu },\varvec{y})}{d\varvec{\mu }_v}=\dfrac{1}{2}\left( \dfrac{d[\varvec{\mu }_{x^{\prime }} -(-\varvec{\mu }_x+\theta _f\varvec{\mu }_v)]^T}{d\varvec{\mu }_v}2\Pi _w\varvec{\varepsilon }_x\right) +\dfrac{1}{2}\left( \dfrac{d(\varvec{\mu }_v)^T}{d\varvec{\mu }_v}2\Pi _v\varvec{\varepsilon }_v\right) =-\theta _f^T\Pi _w\varvec{\varepsilon }_x+\Pi _v\varvec{\varepsilon }_v \end{aligned}$$

where we used the matrix differentiation property

35 $$\begin{aligned} \dfrac{d\varvec{x}^TA\varvec{x}}{d\varvec{z}}=2\dfrac{d\varvec{x}}{d\varvec{z}}A\varvec{x} \end{aligned}$$

for symmmetric $$A$$.

## Supplementary Information

Supplementary Legends.

Supplementary Video 1.

## Data Availability

Code to replicate the simulations is available at https://zenodo.org/record/7941693.

## References

[1] Mauk, M. D. & Ruiz, B. P. Learning-dependent timing of Pavlovian eyelid responses: Differential conditioning using multiple interstimulus intervals. *Behav. Neurosci.* **106**, 666–681 (1992). 1503659 10.1037//0735-7044.106.4.666

[2] Fonio, E. *et al.* Coordination of sniffing and whisking depends on the mode of interaction with the environment. *Isr. J. Ecol. Evol.* **61**, 95–105 (2015).

[3] Kurnikova, A., Moore, J. D., Liao, S.-M., Deschênes, M. & Kleinfeld, D. Coordination of orofacial motor actions into exploratory behavior by rat. *Curr. Biol.* **27**, 688–696 (2017). 28216320 10.1016/j.cub.2017.01.013 PMC5653531

[4] Romano, V. *et al.* Functional convergence of autonomic and sensorimotor processing in the lateral cerebellum. *Cell Rep.* **32**, 107867 (2020). 32640232 10.1016/j.celrep.2020.107867 PMC7351113

[5] Fiocchi, F. R., Dijkhuizen, S., Koekkoek, S. K. E., De Zeeuw, C. I. & Boele, H. J. Stimulus generalization in mice during Pavlovian eyeblink conditioning. *eNeuro* **9**, 25 (2022). 10.1523/ENEURO.0400-21.2022 PMC8941640 35228312

[6] Gao, Z. *et al.* A cortico-cerebellar loop for motor planning. *Nature* **563**, 113–116 (2018). 30333626 10.1038/s41586-018-0633-x PMC6212318

[7] Deverett, B., Koay, S. A., Oostland, M. & Wang, S.S.-H. Cerebellar involvement in an evidence-accumulation decision-making task. *eLife* **7**, e36781 (2018). 30102151 10.7554/eLife.36781 PMC6105309

[8] VinuezaVeloz, M. F. *et al.* Cerebellar control of gait and interlimb coordination. *Brain Struct. Funct.* **220**, 3513–3536 (2015). 25139623 10.1007/s00429-014-0870-1 PMC4575700

[9] Lin, Q. *et al.* Cerebellar neurodynamics predict decision timing and outcome on the single-trial level. *Cell* **180**, 536-551.e17 (2020). 31955849 10.1016/j.cell.2019.12.018 PMC7299501

[10] Baumann, O. *et al.* Consensus paper: The role of the cerebellum in perceptual processes. *Cerebellum* **14**, 197–220 (2015). 25479821 10.1007/s12311-014-0627-7 PMC4346664

[11] Rondi-Reig, L., Paradis, A.-L. & Fallahnezhad, M. A liaison brought to light: Cerebellum-hippocampus, partners for spatial cognition. *Cerebellum* **21**, 826–837 (2022). 35752720 10.1007/s12311-022-01422-3

[12] Kelly, E. *et al.* Regulation of autism-relevant behaviors by cerebellar-prefrontal cortical circuits. *Nat. Neurosci.* **23**, 1102–1110 (2020). 32661395 10.1038/s41593-020-0665-z PMC7483861

[13] Paulin, M. G. A model of the role of the cerebellum in tracking and controlling movements. *Hum. Mov. Sci.* **12**, 5–16 (1993).

[14] Miall, R. C., Weir, D. J., Wolpert, D. M. & Stein, J. F. Is the cerebellum a Smith predictor?. *J. Mot. Behav.* **25**(3), 203–16 (1993). 12581990 10.1080/00222895.1993.9942050

[15] Wolpert, D. M., Miall, R. & Kawato, M. Internal models in the cerebellum. *Trends Cogn. Sci.* **2**, 338–347 (1998). 21227230 10.1016/s1364-6613(98)01221-2

[16] Ito, M. Control of mental activities by internal models in the cerebellum. *Nat. Rev. Neurosci.* **9**, 304–313 (2008). 18319727 10.1038/nrn2332

[17] Therrien, A. S. & Bastian, A. J. Cerebellar damage impairs internal predictions for sensory and motor function. *Curr. Opin. Neurobiol.* **33**, 127–133 (2015). 25863011 10.1016/j.conb.2015.03.013 PMC4786071

[18] Brooks, J. X., Carriot, J. & Cullen, K. E. Learning to expect the unexpected: Rapid updating in primate cerebellum during voluntary self-motion. *Nat. Neurosci.* **18**, 1310–1317 (2015). 26237366 10.1038/nn.4077 PMC6102711

[19] Argyropoulos, G. P. The cerebellum, internal models and prediction in ‘non-motor’ aspects of language: A critical review. *Brain Lang.* **161**, 4–17 (2016). 26320734 10.1016/j.bandl.2015.08.003

[20] Tanaka, H., Ishikawa, T. & Kakei, S. Neural evidence of the cerebellum as a state predictor. *Cerebellum* **18**, 349–371 (2019). 30627965 10.1007/s12311-018-0996-4 PMC6517560

[21] Apps, R. & Hawkes, R. Cerebellar cortical organization: A one-map hypothesis. *Nat. Rev. Neurosci.* **10**, 670–681 (2009). 19693030 10.1038/nrn2698

[22] Sobel, N. *et al.* Odorant-induced and sniff-induced activation in the cerebellum of the human. *J. Neurosci.* **18**, 8990–9001 (1998). 9787004 10.1523/JNEUROSCI.18-21-08990.1998 PMC6793565

[23] Chabrol, F. P., Arenz, A., Wiechert, M. T., Margrie, T. W. & Digregorio, D. A. Synaptic diversity enables temporal coding of coincident multisensory inputs in single neurons. *Nat. Neurosci.* **18**, 718–727 (2015). 25821914 10.1038/nn.3974 PMC4413433

[24] Ishikawa, T., Shimuta, M. & Häusser, M. Multimodal sensory integration in single cerebellar granule cells in vivo. *eLife* **4**, 25 (2015). 10.7554/eLife.12916 PMC4798944 26714108

[25] Wagner, M. J. & Luo, L. Neocortex-cerebellum circuits for cognitive processing. *Trends Neurosci.* **43**, 42–54 (2020). 31787351 10.1016/j.tins.2019.11.002 PMC6942222

[26] D’Angelo, E. The organization of plasticity in the cerebellar cortex: From synapses to control. In *Cerebellar Learning, Vol. 210 of Progress in Brain Research* (ed. Ramnani, N.) 31–58 (Elsevier, 2014). 10.1016/B978-0-444-63356-9.00002-9 24916288

[27] Doya, K. What are the computations of the cerebellum, the basal ganglia and the cerebral cortex?. *Neural Netw.* **12**, 961–974 (1999). 12662639 10.1016/s0893-6080(99)00046-5

[28] Friston, K. & Herreros, I. Active inference and learning in the cerebellum. *Neural Comput.* **28**, 1812–1839 (2016). 27391681 10.1162/NECO_a_00863

[29] Ramnani, N. Automatic and controlled processing in the corticocerebellar system. In *Cerebellar Learning, Vol 210 of Progress in Brain Research* (ed. Ramnani, N.) 255–285 (Elsevier, USA, 2014). 10.1016/B978-0-444-63356-9.00010-8 24916296

[30] Shadmehr, R. & Krakauer, J. W. A computational neuroanatomy for motor control. *Exp. Brain Res.* **185**, 359–381 (2008). 18251019 10.1007/s00221-008-1280-5 PMC2553854

[31] Friston, K. The free-energy principle: A rough guide to the brain?. *Trends Cogn. Sci.* **13**, 293–301 (2009). 19559644 10.1016/j.tics.2009.04.005

[32] Friston, K. The free-energy principle: A unified brain theory?. *Nat. Rev. Neurosci.* **11**, 127–138 (2010). 20068583 10.1038/nrn2787

[33] Ramstead, M. J. D. *et al.* On Bayesian mechanics: A physics of and by beliefs (2022). 10.1098/rsfs.2022.0029 PMC10198254 37213925

[34] Palacios, E. R., Isomura, T., Parr, T. & Friston, K. The emergence of synchrony in networks of mutually inferring neurons. *Sci. Rep.* **9**, 6412 (2019). 31040386 10.1038/s41598-019-42821-7 PMC6491596

[35] Friston, K. & Kiebel, S. Predictive coding under the free-energy principle. *Philos. Trans. R. Soc. B Biol. Sci.* **364**, 1211–1221 (2009). 10.1098/rstb.2008.0300 PMC2666703 19528002

[36] Kiebel, S. J., von Kriegstein, K., Daunizeau, J. & Friston, K. J. Recognizing sequences of sequences. *PLoS Comput. Biol.* **5**, 1–13 (2009). 10.1371/journal.pcbi.1000464 PMC2714976 19680429

[37] Kelso, J. A. S. Unifying large- and small-scale theories of coordination. *Entropy* **23**, 2 (2021). 10.3390/e23050537 PMC8146522 33925736

[38] Friston, K., Trujillo-Barreto, N. & DEM Daunizeau, J. A variational treatment of dynamic systems. *Neuroimage* **41**, 849–885 (2008). 18434205 10.1016/j.neuroimage.2008.02.054

[39] Buckley, C. L., Kim, C. S., McGregor, S. & Seth, A. K. The free energy principle for action and perception: A mathematical review. *J. Math. Psychol.* **81**, 55–79 (2017).

[40] Hohwy, J. The self-evidencing brain. *Noûs* **50**, 259–285 (2016).

[41] Mariën, P. & Borgatti, R. Chapsster 11-language and the cerebellum. In *The Cerebellum: From Embryology to Diagnostic Investigations, Vol 154 of Handbook of Clinical Neurology* (eds Manto, M. & Huisman, T. A.) 181–202 (Elsevier, 2018). 10.1016/B978-0-444-63956-1.00011-4 29903439

[42] Straub, I. *et al.* Gradients in the mammalian cerebellar cortex enable Fourier-like transformation and improve storing capacity. *eLife* **9**, e51771 (2020). 32022688 10.7554/eLife.51771 PMC7002074

[43] Palacios, E. R., Houghton, C. & Chadderton, P. Accounting for uncertainty: Inhibition for neural inference in the cerebellum. *Proc. R. Soc. B Biol. Sci.* **288**, 20210276 (2021). 10.1098/rspb.2021.0276 PMC8059656 33757352

[44] Houck, B. D. & Person, A. L. Cerebellar premotor output neurons collateralize to innervate the cerebellar cortex. *J. Comp. Neurol.* **523**, 2254–2271 (2015). 25869188 10.1002/cne.23787 PMC4537674

[45] Ankri, L. *et al.* A novel inhibitory nucleo-cortical circuit controls cerebellar Golgi cell activity. *eLife* **4**, e06262 (2015). 25965178 10.7554/eLife.06262 PMC4461794

[46] Witter, L., Rudolph, S., Pressler, R. T., Lahlaf, S. I. & Regehr, W. G. Purkinje cell collaterals enable output signals from the cerebellar cortex to feed back to Purkinje cells and interneurons. *Neuron* **91**, 312–319 (2016). 27346533 10.1016/j.neuron.2016.05.037 PMC4969194

[47] Guo, C. *et al.* Purkinje cells directly inhibit granule cells in specialized regions of the cerebellar cortex. *Neuron* **91**, 1330–1341 (2016). 27593180 10.1016/j.neuron.2016.08.011 PMC5853127

[48] Coesmans, M., Weber, J. T., De Zeeuw, C. I. & Hansel, C. Bidirectional parallel fiber plasticity in the cerebellum under climbing fiber control. *Neuron* **44**, 691–700 (2004). 15541316 10.1016/j.neuron.2004.10.031

[49] Yang, Y. & Lisberger, S. G. Interaction of plasticity and circuit organization during the acquisition of cerebellum-dependent motor learning. *eLife* **2**, e01574 (2013). 24381248 10.7554/eLife.01574 PMC3871706

[50] Bellingham, M. C. Driving respiration: The respiratory central pattern generator. *Clin. Exp. Pharmacol. Physiol.* **25**, 847–856 (1998). 9784928 10.1111/j.1440-1681.1998.tb02166.x

[51] Takatoh, J. *et al.* The whisking oscillator circuit. *Nature* **609**, 560–568 (2022). 36045290 10.1038/s41586-022-05144-8 PMC10038238

[52] Friston, K. What is optimal about motor control?. *Neuron* **72**, 488–498 (2011). 22078508 10.1016/j.neuron.2011.10.018

[53] Chen, S., Augustine, G. J. & Chadderton, P. The cerebellum linearly encodes whisker position during voluntary movement. *eLife* **5**, 25 (2016). 10.7554/eLife.10509 PMC4737656 26780828

[54] Hong, S. *et al.* Multiplexed coding by cerebellar Purkinje neurons. *eLife* **5**, e13810 (2016). 27458803 10.7554/eLife.13810 PMC4961467

[55] Raymond, J. L. & Medina, J. F. Computational principles of supervised learning in the cerebellum. *Annu. Rev. Neurosci.* **41**, 233–253 (2018). 29986160 10.1146/annurev-neuro-080317-061948 PMC6056176

[56] Machado, A. S., Darmohray, D. M., Fayad, J., Marques, H. G. & Carey, M. R. A quantitative framework for whole-body coordination reveals specific deficits in freely walking ataxic mice. *eLife* **4**, e07892 (2015). 26433022 10.7554/eLife.07892 PMC4630674

[57] Fu, Q. G., Mason, C. R., Flament, D., Coltz, J. D. & Ebner, T. J. Movement kinematics encoded in complex spike discharge of primate cerebellar Purkinje cells. *NeuroReport* **8**, 523–529 (1997). 9080441 10.1097/00001756-199701200-00029

[58] Ju, C. *et al.* Neurons of the inferior olive respond to broad classes of sensory input while subject to homeostatic control. *J. Physiol.* **597**, 2483–2514 (2019). 30908629 10.1113/JP277413 PMC6487939

[59] Heffley, W. & Hull, C. Classical conditioning drives learned reward prediction signals in climbing fibers across the lateral cerebellum. *Elife* **8**, 25 (2019). 10.7554/eLife.46764 PMC6845228 31509108

[60] Ikezoe, K. *et al.* Cerebellar climbing fibers convey behavioral information of multiplex modalities and form functional modules. *BioRxiv* **20**, 20 (2022).

[61] Perrinet, L. U., Adams, R. A. & Friston, K. J. Active inference, eye movements and oculomotor delays. *Biol. Cybern.* **108**, 777–801 (2014). 25128318 10.1007/s00422-014-0620-8 PMC4250571

[62] Guell, X., Gabrieli, J. D. & Schmahmann, J. D. Triple representation of language, working memory, social and emotion processing in the cerebellum: Convergent evidence from task and seed-based resting-state fmri analyses in a single large cohort. *Neuroimage* **172**, 437–449 (2018). 29408539 10.1016/j.neuroimage.2018.01.082 PMC5910233

[63] Kostadinov, D., Beau, M., Blanco-Pozo, M. & Häusser, M. Predictive and reactive reward signals conveyed by climbing fiber inputs to cerebellar Purkinje cells. *Nat. Neurosci.* **22**, 950–962 (2019). 31036947 10.1038/s41593-019-0381-8 PMC7612392

[64] Wagner, M. J. *et al.* Shared cortex-cerebellum dynamics in the execution and learning of a motor task. *Cell* **177**, 669-682.e24 (2019). 30929904 10.1016/j.cell.2019.02.019 PMC6500577

[65] Lanore, F., Cayco-Gajic, N. A., Gurnani, H., Coyle, D. & Silver, R. A. Cerebellar granule cell axons support high-dimensional representations. *Nat. Neurosci.* **24**, 1142–1150 (2021). 34168340 10.1038/s41593-021-00873-x PMC7611462

[66] Titley, H. K. *et al.* Intrinsic excitability increase in cerebellar Purkinje cells after delay eye-blink conditioning in mice. *J. Neurosci.* **40**, 2038–2046 (2020). 32015022 10.1523/JNEUROSCI.2259-19.2019 PMC7055141

[67] ten Brinke, M. M. *et al.* Dynamic modulation of activity in cerebellar nuclei neurons during pavlovian eyeblink conditioning in mice. *eLife* **6**, e28132 (2017). 29243588 10.7554/eLife.28132 PMC5760204

[68] Bogacz, R. A tutorial on the free-energy framework for modelling perception and learning. *J. Math. Psychol.* **76**, 198–211 (2017). 28298703 10.1016/j.jmp.2015.11.003 PMC5341759

[69] Friston, K. Hierarchical models in the brain. *PLoS Comput. Biol.* **4**, 1–24 (2008). 10.1371/journal.pcbi.1000211 PMC2570625 18989391

[70] Sathyamurthy, A. *et al.* Cerebellospinal neurons regulate motor performance and motor learning. *Cell Rep.* **31**, 107595 (2020). 32402292 10.1016/j.celrep.2020.107595 PMC7263484

[71] Watson, T. C. *et al.* Anatomical and physiological foundations of cerebello-hippocampal interaction. *eLife* **8**, e41896 (2019). 31205000 10.7554/eLife.41896 PMC6579515

[72] Bostan, A. C. & Strick, P. L. The basal ganglia and the cerebellum: Nodes in an integrated network. *Nat. Rev. Neurosci.* **19**, 338–350 (2018). 29643480 10.1038/s41583-018-0002-7 PMC6503669

[73] Palesi, F. *et al.* Contralateral cortico-ponto-cerebellar pathways reconstruction in humans in vivo: Implications for reciprocal cerebro-cerebellar structural connectivity in motor and non-motor areas. *Sci. Rep.* **7**, 12841 (2017). 28993670 10.1038/s41598-017-13079-8 PMC5634467

[74] Becker, M. I. & Person, A. L. Cerebellar control of reach kinematics for endpoint precision. *Neuron* **103**, 335-348.e5 (2019). 31174960 10.1016/j.neuron.2019.05.007 PMC6790131

[75] Shemesh, A. A. & Zee, D. S. Eye movement disorders and the cerebellum. *J. Clin. Neurophysiol.* **36**, 25 (2019). 31688323 10.1097/WNP.0000000000000579 PMC6986321

[76] Nashef, A., Cohen, O., Harel, R., Israel, Z. & Prut, Y. Reversible block of cerebellar outflow reveals cortical circuitry for motor coordination. *Cell Rep.* **27**, 2608-2619.e4 (2019). 31141686 10.1016/j.celrep.2019.04.100

