# Author Correction: Cerebellar state estimation enables resilient coupling across behavioural domains

**DOI:** 10.1038/s41598-024-79469-x

**Published:** 2024-11-20

**Authors:** Ensor Rafael Palacios, Paul Chadderton, Karl Friston, Conor Houghton

**Affiliations:** 1 School of Physiology Pharmacology and Neuroscience, University of Bristol, Bristol, BS8 1TD UK; 2 Wellcome Centre for Human Neuroimaging, UCL, London, WC1N 3AR UK; 3 Department of Computer Science, University of Bristol, Bristol, BS8 1UB UK

Correction to: *Scientific reports* 10.1038/s41598-024-56811-x, published online 19 March 2024

The Funding section in the original version of this Article was omitted. The Funding section now reads:

“Funding for this research was provided by the Wellcome Trust (220114/Z/20/Z, 209453/Z/17/Z, 205103/Z/16/Z, 108899/B/15/Z), the Canada-UK Artificial Intelligence Initiative (ES/T01279X/1) and the Leverhulme Trust (RF-2021-533).”

The original Article has been corrected.

