# Supplementary material for: Cerebellar state estimation enables resilient coupling across behavioural domains
Source: Sci Rep. 2024 Mar 19;14:6641. doi: 10.1038/s41598-024-56811-x (PMC10951354; doi:10.1038/s41598-024-56811-x)
Supplement: Supplementary file 1 — Supplementary Legends. [file 41598_2024_56811_MOESM1_ESM.pdf]

# Cerebellar state estimation enables resilient coupling across behavioural domains - Supplementary information

Ensor Rafael Palacios Paul Chadderton Karl Friston Conor Houghton

**Video S1. Combining intrinsic offset and noise conditions** Dynamical visualisation of whisking and respiration (top) and their difference in the time domain (middle) and in the joint state space (bottom). The cerebellum expects coordination between whisking and respiration, and is able to coordinate the two behaviours despite the presence of independent gaussian noise added to their dynamics and an offset in their angular velocities. During an intermediate period, however, we simulate transient inactivation of the **CN** output to extra-cerebellar regions; consequently, whisking and respiration desynchronise. Blue and red colors indicate periods when the cerebellum does and does not couple back to extra-cerebellar structures, respectively.
